# Multi-omic analyses identify molecular targets of Chd7 that contribute to CHARGE syndrome model phenotypes

**DOI:** 10.1242/dmm.052592

**Published:** 2026-03-31

**Authors:** Melody B. Hancock, Dana R. Ruby, Rachael A. Bieler, D. Chris Cole, Kurt C. Marsden

**Affiliations:** Department of Biological Sciences, North Carolina State University, Raleigh, NC 27607, USA

**Keywords:** CHARGE syndrome, CHD7, Developmental disorder, Transcriptomics, Proteomics, Behavior

## Abstract

CHARGE syndrome is a developmental disorder that affects 1 in 10,000 births, and patients exhibit both physical and behavioral characteristics. *De novo* variants in chromodomain helicase DNA binding protein 7 (*CHD7*) cause 67% of CHARGE syndrome cases. CHD7 is a DNA-binding chromatin remodeler with thousands of predicted binding sites in the genome, making it challenging to define molecular pathways linking loss of *CHD7* to CHARGE syndrome phenotypes. To address this problem, here, we used a previously characterized zebrafish model of CHARGE syndrome to generate transcriptomic and proteomic datasets from larval zebrafish head tissue at two developmental time points. By integrating these datasets with differential expression, pathway and upstream regulator analyses, we identified multiple consistently dysregulated pathways and defined a set of candidate genes that link loss of *chd7* with disease-related phenotypes. Finally, we identified that CRISPR/Cas9-mediated knockdown of *capgb*, *nefla* or *rdh5* phenocopies behavioral defects seen in *chd7* mutants, functionally validating the roles of these genes. Our data provide a resource for further investigation of molecular mediators of CHD7 and a template to reveal functionally relevant therapeutic targets to alleviate specific aspects of CHARGE syndrome.

## Teaser

**Summary:** Using transcriptomic and proteomic analysis of *chd7* wild-type, heterozygous and homozygous mutant zebrafish brain tissue, we identified Chd7 target genes – *capgb*, *nefla* and *rdh5* – that contribute to phenotypes in a model of CHARGE syndrome.

## INTRODUCTION

CHARGE syndrome is an autosomal dominant developmental condition that occurs in 1 in 10,000 newborns (Blake et al., 1998) and is named for its previous diagnostic criteria: coloboma (‘C’), heart disease (‘H’), atresia choanae (‘A’), retardation of growth and development and/or CNS anomalies (‘R’), genital hypoplasia (‘G’) and ear anomalies and/or deafness (‘E’) (Pagon et al., 1981). Affected individuals display physical and behavioral characteristics such as autistic-like behaviors (Hartshorne et al., 2005), obsessive compulsive disorder (Hartshorne et al., 2017), attention-deficit/hyperactivity disorder (Hartshorne and Cypher, 2004), anxiety (Hartshorne et al., 2017), aggression (Blake et al., 2005), intellectual disability (Wachtel et al., 2007) and sensory disorders (Edwards et al., 1995).

Sixty-seven percent of CHARGE syndrome cases can be attributed to variants in chromodomain helicase DNA binding protein 7 (*CHD7*) (Zentner et al., 2010). *CHD7* is ubiquitously expressed in early human development (Sanlaville et al., 2006), a pattern conserved across species including mouse (Bosman et al., 2005), zebrafish (Patten et al., 2012), fly (Daubresse et al., 1999) and chick (Williams et al., 2024). CHD7 is part of the CHD family of chromatin remodelers and is recruited to specific histone modifications and enhancer regions to regulate DNA accessibility via ATP-dependent chromatin remodeling (Basson and van Ravenswaaij-Arts, 2015). CHD7 chromatin immunoprecipitation sequencing (ChIP-seq) experiments have found 10,000 predicted binding sites in mouse embryonic stem cells (Schnetz et al., 2010) and 9000 predicted binding sites in mouse cerebellar granule cell precursors (Reddy et al., 2021). Chd7 is known to be important for neurodevelopment through its roles in neural crest cell migration and specification (Schulz et al., 2014b), neurogenesis (Feng et al., 2013), neuronal differentiation (Jones et al., 2015), and activation of neural crest transcription factors *sox9*, *twist* and *slug* (Bajpai et al., 2010). When embryonic stem cells transition to neural progenitors, CHD7 binding patterns change, suggesting that the functions of CHD7 shift across neurodevelopmental stages (Schnetz et al., 2009).

Models of CHARGE syndrome have been established in mouse, fly and zebrafish, and these have been studied to determine how loss of *chd7* affects multiple systems: brain defects (Whittaker et al., 2017b; Feng et al., 2017; Donovan et al., 2017), ear defects (Bosman et al., 2005; Gao et al., 2024), eye defects (Gage et al., 2015), cardiac defects (Bergman et al., 2010; Bosman et al., 2005) and craniofacial defects (Asad et al., 2016; Asad and Sachidanandan, 2020; Balow et al., 2013). These models have also shown how loss of *chd7* affects sensory systems (Bergman et al., 2010; Layman et al., 2009; Bosman et al., 2005), behavior (Hodorovich et al., 2023; Jamadagni et al., 2021) and the transcriptome (Jamadagni et al., 2021; Yao et al., 2020; Stathopoulou et al., 2023; Huang et al., 2025). Despite these advances in understanding the biological roles of *chd7*, the molecular mechanisms that directly link loss of *CHD7* with CHARGE syndrome phenotypes remain unclear.

In this study, we aimed to leverage the advantages of our zebrafish model of CHARGE syndrome (Hodorovich et al., 2023) to investigate the molecular pathophysiology of neurobehavioral symptoms associated with CHARGE syndrome. By integrating transcriptomic and proteomic analyses across two developmental timepoints, we identified key *chd7*-dependent genes, proteins and pathways, including histone modification, GABAergic receptors, NMDA receptors, ROBO receptors and SNARE signaling. Finally, we filtered our datasets to identify individual candidate mediators that were consistently dysregulated at the RNA and protein level, at both timepoints, and in both *chd7* heterozygotes and homozygous mutants. We cross-referenced these with known CHD7 binding targets, and, using CRISPR/Cas9-mediated knockdown, we functionally validated *capping actin protein gelsolin like b* (*capgb*), *neurofilament light chain a* (*nefla*) and *retinol dehydrogenase 5* (*rdh5*) as likely contributors to CHARGE model behavioral and morphological phenotypes.

## RESULTS

To identify molecular mediators of Chd7-dependent development, we collected head tissue at 3 and 5 days post fertilization (dpf) from zebrafish larvae derived from incrosses of *chd7* heterozygotes. These timepoints represent the earliest developmental stages at which CHARGE syndrome-related morphological and behavioral phenotypes emerge in both *chd7* heterozygous and mutant larvae, with higher penetrance and severity in mutants (Hodorovich et al., 2023). By analyzing both timepoints, we also sought to gain insight into how Chd7 modulates developmental gene expression patterns across these stages. Tail tissue was used for genotyping, and head tissue from larvae (*n*=20-30 per genotype) was pooled for RNA or protein isolation to generate four biological replicates for all three genotypes: *chd7* wild-type (WT), heterozygous (HT) and homozygous mutant (MUT) samples. Following RNA sequencing and mass spectrometry using standard procedures (see Materials and Methods), we performed differential expression analysis comparing HT to WT samples (HT versus WT) and MUT to WT samples (MUT versus WT) to identify altered gene and protein levels due to loss of *chd7*.

**Fig. 1.**
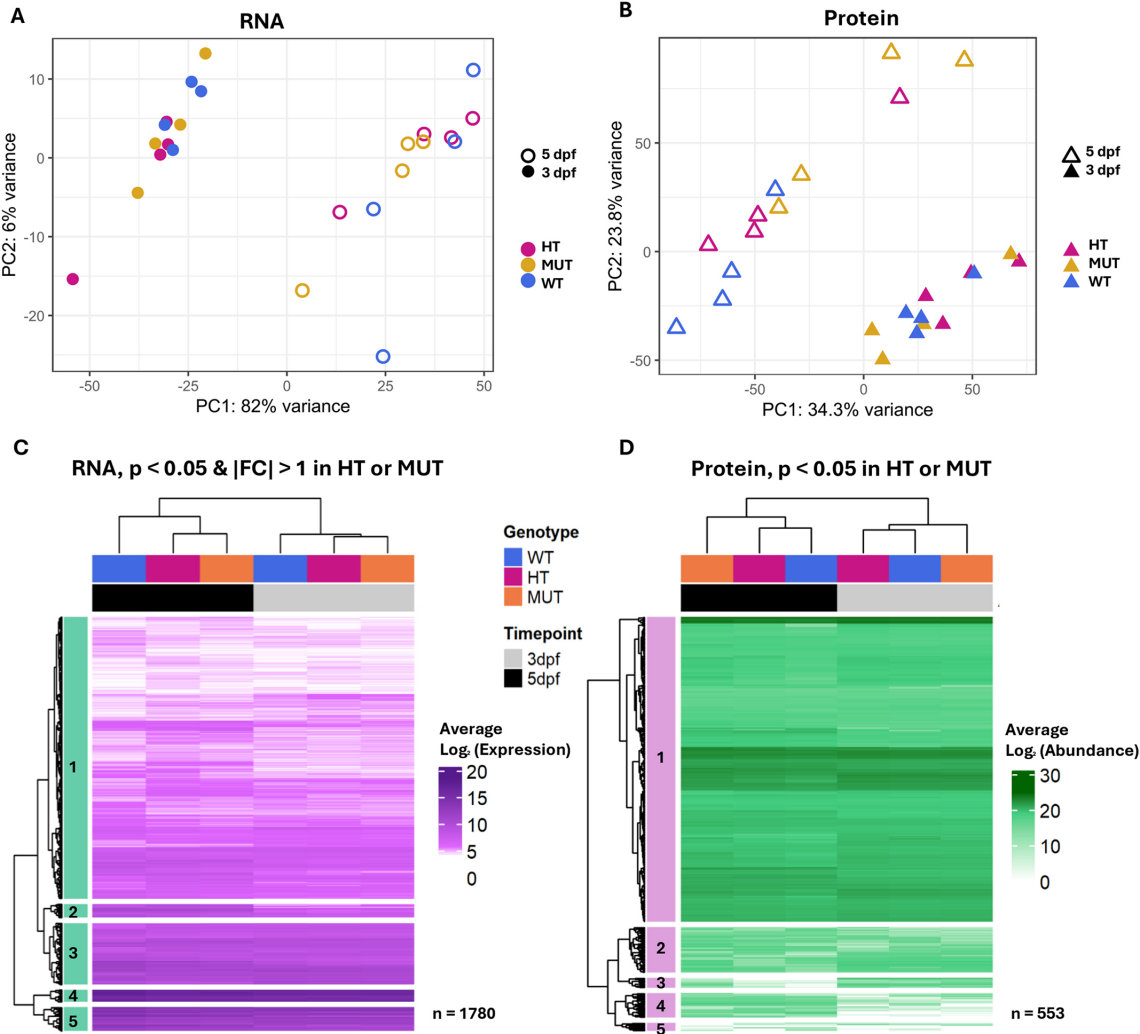
**Loss of *chd7* causes dysregulation of genes associated with neurodevelopmental and transcriptional regulation functions.** (A,B) Principal component (PC) analysis of quantified RNAs for each sample and replicate (A), and quantified proteins for each sample and replicate (B). (C,D) Heatmaps of Log_2_ expression averaged across biological replicates with hierarchical clustering by similar expression patterns of differentially expressed genes (DEGs) with *P*<0.05 (Wald test) and absolute value of fold change (FC)>1 in heterozygous (HT) or homozygous mutant (MUT) versus wild-type (WT) comparisons (*n*=1780) (C), and differentially expressed proteins (DEPs) with *P*<0.05 (one-way ANOVA) (D) in HT or MUT versus WT comparisons (*n*=553).

First, to determine the degree of similarity in overall RNA and protein expression between replicates, timepoints and genotypes, we used principal component analysis (PCA). PCA of the transcriptome data showed that samples from all genotypes clustered by timepoint, and principal component (PC)1 alone accounted for 82% of the variance between samples (Fig. 1A). PCA of the proteome data revealed similar clustering by timepoint, with PC1 accounting for 34.4% and PC2 23.8% of the variance (Fig. 1B). Although RNA samples showed little clustering by *chd7* genotype, protein samples did cluster somewhat by genotype, particularly at 5 dpf. Overall, developmental changes in gene expression from 3 to 5 dpf were the primary drivers of the differences between samples; but, by 5 dpf, Chd7-dependent molecular changes could be seen by PCA.

Next, we used hierarchical clustering to find genes with similar expression patterns across timepoints and genotypes. We first identified significantly differentially expressed genes/RNAs (DEGs) and differentially expressed proteins (DEPs) in HT and MUT samples compared to WT, and we then generated heatmaps to visualize expression of these DEGs. We plotted average Log_2_ transformed expression of RNAs (Fig. 1C) and average Log_2_ transformed abundances of proteins (Fig. 1D) significant in either HT versus WT or MUT versus WT comparisons and performed k-means hierarchical clustering by Euclidean distance (Gu, 2022). Heatmaps of Log_2_ transformed expression and abundance across biological replicates (not averaged) can be found in Figs S1 and S2. DEGs and DEPs again clustered first by timepoint, with similar overall expression patterns in HT and MUT compared to WT. DEGs in cluster 1 included 1277 unique RNAs significantly [false discovery rate (FDR<0.05)] associated with Gene Ontology (GO) biological processes detection of stimulus (GO:0051606), sensory perception (GO:0007600) and chromatin remodeling (GO:0006338) (Table S1). DEGs in cluster 5 included 54 unique RNAs significantly (FDR<0.05) associated with GO biological processes visual system development (GO:0150063), visual perception (GO:0007601) and sensory perception of light stimulus (GO:0050953) (Table S5). DEPs in cluster 1 included 403 unique proteins significantly (FDR<0.05) associated with GO biological processes gene expression (GO:0010467), mRNA processing (GO:0006397) and RNA splicing (GO:0008380) (Table S1). Lists of DEGs and DEPs from each cluster and GO biological processes are provided in Tables S1-S5.

In summary, we found that loss of either one or two copies of *chd7* produces globally similar changes in RNA expression patterns. Protein expression, however, was more similar in WT and HT larvae, suggesting that translation regulation may be more strongly impacted in *chd7* MUT than in HT larvae.

### *chd7* indirectly regulates developmental RNA-processing and translation pathways

**Fig. 2.**
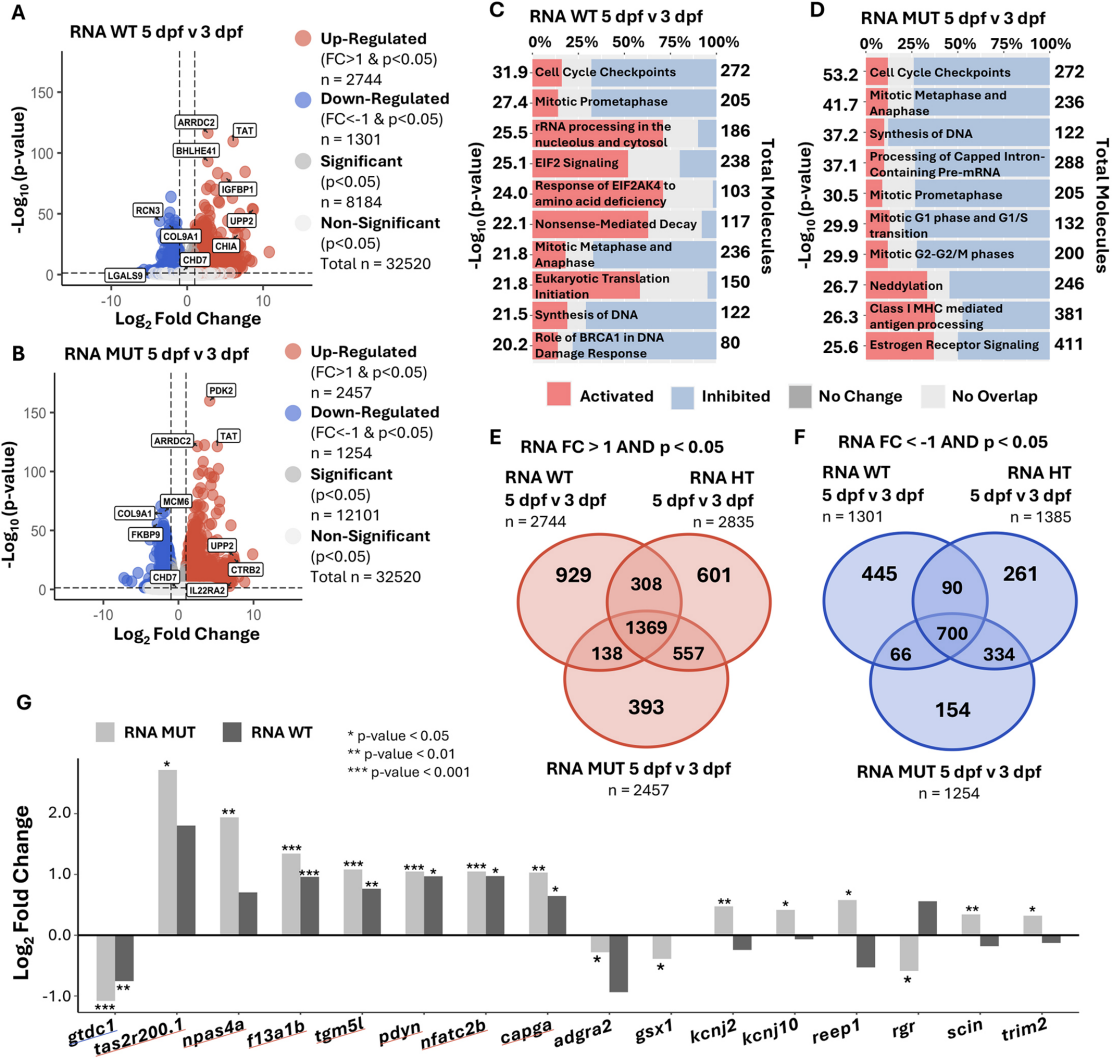
**Neural-related transcripts with protein counterparts change developmentally from 3 dpf to 5 dpf due to loss of *chd7.*** (A,B) Volcano plots of DEGs: 5 dpf WT compared to 3 dpf WT samples (A), and 5 dpf MUT compared to 3 dpf MUT samples (B). (C,D) Ingenuity Pathway Analysis (IPA) pathway enrichment by patterns of DEGs from 5 dpf WT compared to 3 dpf WT samples (C), and 5 dpf MUT compared to 3 dpf MUT samples (D). (E,F) Overlap of DEGs in 5 dpf WT versus 3 dpf WT, 5 dpf HT versus 3 dpf HT, and 5 dpf MUT versus 3 dpf MUT. (E) DEGs upregulated with FC>1 and *P*<0.05 (Wald test). (F) DEGs downregulated with FC<−1 and *P*<0.05 (Wald test). (G) Log_2_ FC for DEGs selected for most differing FC from Fig. S6, and all DEGs overlapping with the Neuro-GO list in Fig. S7 (underlined in red) and Fig. S8 (underlined in blue). *P<0.05, **P<0.01, ***P<0.001.

To define how *chd7* contributes to normal developmental changes in gene expression, we compared transcriptomes from 5 dpf to 3 dpf within each genotype – WT, HT and MUT. First, we defined DEGs by comparing 5 dpf samples to their corresponding 3 dpf samples and made volcano plots to visualize the distribution of upregulated and downregulated genes across this timeframe (Fig. 2A,B; Fig. S3). We captured 32,530 RNAs in the WT, HT and MUT samples, and, in all three genotypes, more than twice as many genes were upregulated than downregulated at 5 dpf (Fig. 2A,B; Fig. S3). We then used Ingenuity Pathway Analysis (IPA) to determine canonical pathways that were differentially regulated in each *chd7* genotype (Tables S7-S9). Multiple cell cycle regulatory pathways were similarly altered in all genotypes (Fig. 2C,D; Fig. S4), indicating that these are independent of *chd7*. We also observed substantial activation of multiple RNA and translational regulation pathways in WT – such as nonsense-mediated decay, eukaryotic translation initiation and EIF2 signaling (Fig. 2C) – that was not seen in *chd7* HT or MUT, suggesting that *chd7* plays an indirect role in translation by regulating transcript levels of key molecules in these pathways. This is further supported by our observation that in HT and MUT – but not WT – processing of capped intron-containing pre-mRNA was strongly inhibited (Fig. 2D; Fig. S4). This analysis also uncovered significant dysregulation of estrogen signaling from 3 to 5 dpf in *chd7* HT and MUT but not WT samples (Fig. 2D; Fig. S4), and although hypogonadism in both sexes is commonly seen in CHARGE syndrome (Pinto et al., 2005), because our samples were enriched for brain tissue, this finding may indicate a role for *chd7* in estrogen-mediated brain development.

We next created Venn diagrams to visualize the differences in developmental changes in gene expression between genotypes. Overall (Fig. S5), we saw more overlap between HT and MUT than between either HT or MUT and WT. We observed a similar pattern when we plotted only genes that were upregulated (Fig. 2E) or downregulated (Fig. 2F) at 5 dpf. Hypothesizing that important *chd7*-dependent neurodevelopmental genes would have uniquely disrupted patterns of expression from 3 to 5 dpf in *chd7* null mutants, we isolated the MUT DEGs that did not overlap with WT or HT and filtered for neuro-related genes using a list of neuro-related genes from GO Accession terms and Ensembl (Table S6). We then compared expression levels of these genes in MUT and WT samples (Fig. 2G; Fig. S6). Many of these neuro-related DEGs were upregulated or downregulated in the same direction in WT and MUT samples, but several were changed in the opposite direction. *Potassium inwardly rectifying channel subfamily J member 2* (*kcnj2*), *potassium inwardly rectifying channel subfamily J member 10* (*kcnj10*), *receptor accessory protein 1* (*reep1*), *scinderin* (*scin*) and *tripartite motif containing 2* (*trim2*) are upregulated in MUT samples and downregulated in WT samples, whereas *retinal G protein coupled receptor* (*rgr*) is downregulated in the MUT sample and upregulated in the WT sample (Fig. 2G; Figs S7 and S8).

Together, these analyses of developmental changes in gene expression reveal that genomic regulation of many developmental processes proceed normally without *chd7*, but neural-related genes – including *kcnj2*, *kcnj10*, *reep1*, *scin*, *trim2* and *rgr* – have differing expression patterns in MUT and WT samples and may contribute to Chd7-dependent neurodevelopment. Furthermore, loss of one or two copies of *chd7* results in general inhibition of canonical pathways compared to WT canonical pathway enrichment.

### Loss of *chd7* causes bidirectional disruption of multiple key signaling pathways in early neurodevelopment

**Fig. 3.**
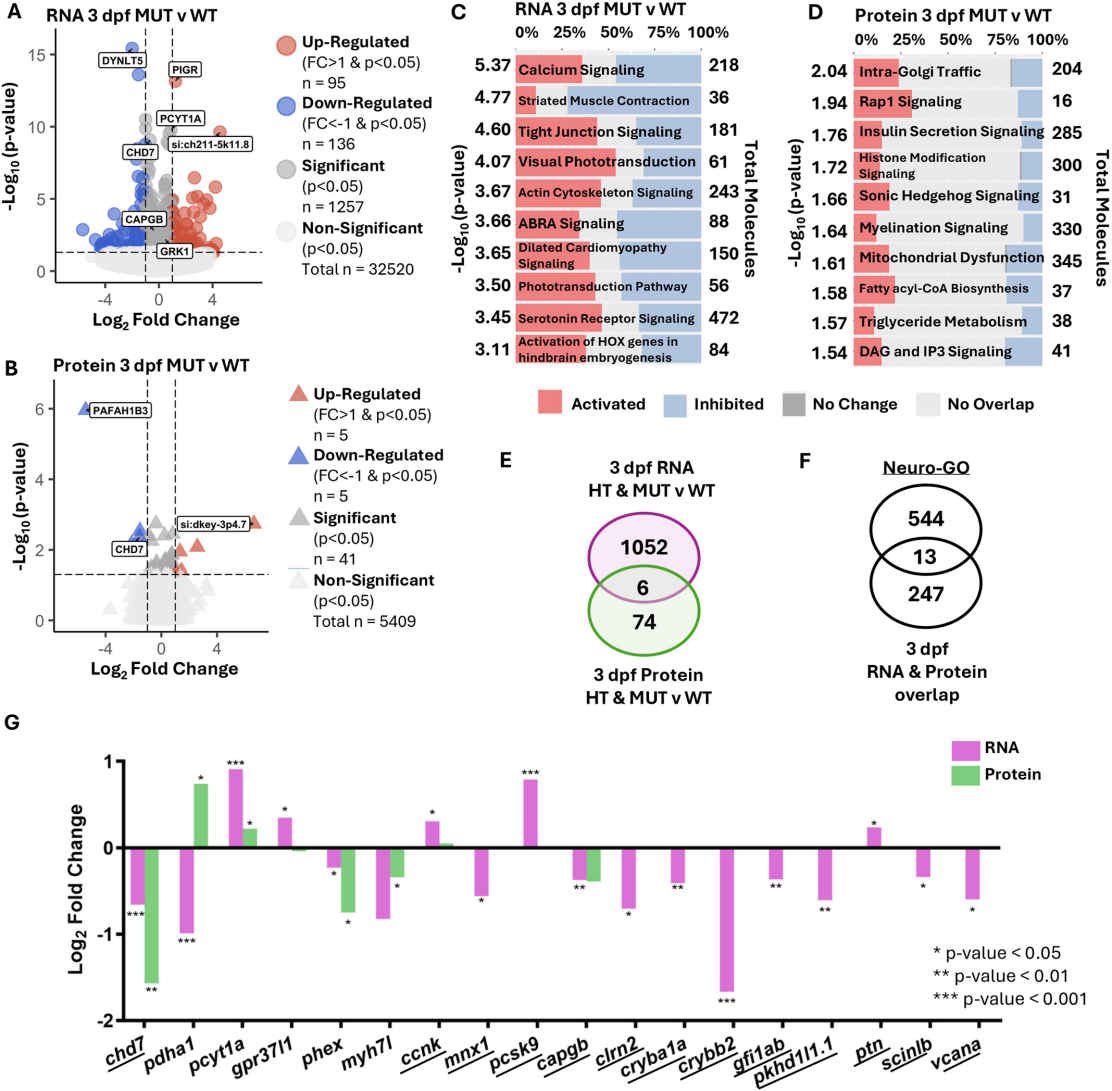
**Early neurodevelopmental transcripts with protein counterparts and neural signaling pathways emerge due to loss of *chd7* at 3 dpf.** (A) Volcano plot of DEGs comparing 3 dpf HT to 3 dpf WT samples. (B) Volcano plot of differentially expressed proteins (DEPs) comparing 3 dpf MUT to 3 dpf WT samples. (C,D) IPA pathway enrichment by patterns of DEGs from 3 dpf MUT compared to WT samples (C), and patterns of DEPs from 3 dpf MUT compared to WT samples (D). (E) Overlap of DEGs with *P*<0.05 (Wald test) from 3 dpf HT and MUT versus WT samples (purple) and DEPs with *P*<0.05 (one-way ANOVA) from 3 dpf HT and MUT versus WT samples (green). (F) Overlap of 3 dpf DEGs and DEPs from Fig. S11 and Neuro-GO list. (G) Log_2_ FC for all DEGs and DEPs from MUT versus WT comparison from overlap in E and from overlap in F (underlined). *P<0.05, **P<0.01, ***P<0.001.

To more directly examine how *chd7* regulates gene and protein expression during early neurodevelopment, we compared 3 dpf RNA and protein levels in HT and MUT samples to those in WT samples. We identified a set of 1091 DEGs and 45 DEPs in *chd7* HT samples and 1257 DEGs and 41 DEPs in *chd7* MUT samples (Fig. 3A,B; Fig. S9). Confirming the validity of our CHARGE model, we found that *chd7* RNA and protein were both significantly downregulated in MUT samples. Similar numbers of transcripts and proteins were upregulated and downregulated in HT and MUT samples. We then used IPA to find canonical pathways with significantly altered enrichment patterns in each genotype (Fig. 3C,D; Fig. S10 and Tables S10-S13). Several pathways were impacted in both HT and MUT samples at the RNA level, including calcium signaling, striated muscle contraction and actin cytoskeleton signaling. We did not find pathways significantly altered in both HT and MUT samples at the protein level, likely because we found many fewer DEPs than DEGs. The only canonical pathway that was disrupted at both the RNA and protein level was SNARE signaling in HT samples. In addition to calcium, SNARE and actin signaling, we found that multiple neural-related molecular pathways were disrupted in HT or MUT samples at 3 dpf: visual phototransduction, serotonin receptor signaling, synaptogenesis signaling, activation of NMDA receptors, myelination signaling, synaptic long-term potentiation and GABAergic receptor signaling (Fig. 3C,D; Fig. S10). Together, these impacts indicate broad disruption of neural development in the absence of *chd7*.

To identify individual genes that may mediate the effects of Chd7, we created Venn diagrams to highlight genes that were consistently dysregulated at both RNA and protein levels (Fig. 3E,F; Fig. S11). We found six transcripts with protein counterparts that were significantly different (*P*<0.05) at 3 dpf in either HT or MUT samples (Fig. 3E). None of these six transcripts overlapped with the Neuro-GO list (Fig. S12). We also found 13 neuro-related transcripts with protein counterparts that were significantly differentially expressed (*P*<0.05) at either the transcript or protein level (Fig. 3F,G). We plotted both transcript and protein expression for these genes and found that most were consistently upregulated or downregulated as both RNA and protein, with the exception of *pyruvate dehydrogenase E1 subunit alpha 1a* (*pdha1a*), for which the transcript was downregulated and protein was upregulated (Fig. 3G). *chd7*, *phosphate regulating endopeptidase X-linked* (*phex*), *myosin heavy chain 7-like* (*myh7l*) and *capgb* were all downregulated at both the RNA and protein level, whereas *phosphate cytidylyltransferase 1A choline* (*pcyt1a*) was upregulated at both the RNA and protein level (Fig. 3G).

These data reinforce previous findings that RNA and protein levels do not always correlate (Nie et al., 2006) and that *chd7* has bidirectional effects on gene expression (Huang et al., 2025; Jamadagni et al., 2021; Stathopoulou et al., 2023; Yao et al., 2020). Furthermore, we found that *chd7* regulates multiple neural signaling pathways and that *capgb*, owing to its consistent dysregulation at RNA and protein levels and across *chd7* genotypes, may be a key effector of *chd7* in neural development.

### Loss of *chd7* disrupts RNA processing and ROBO receptor signaling in 5 dpf zebrafish

**Fig. 4.**
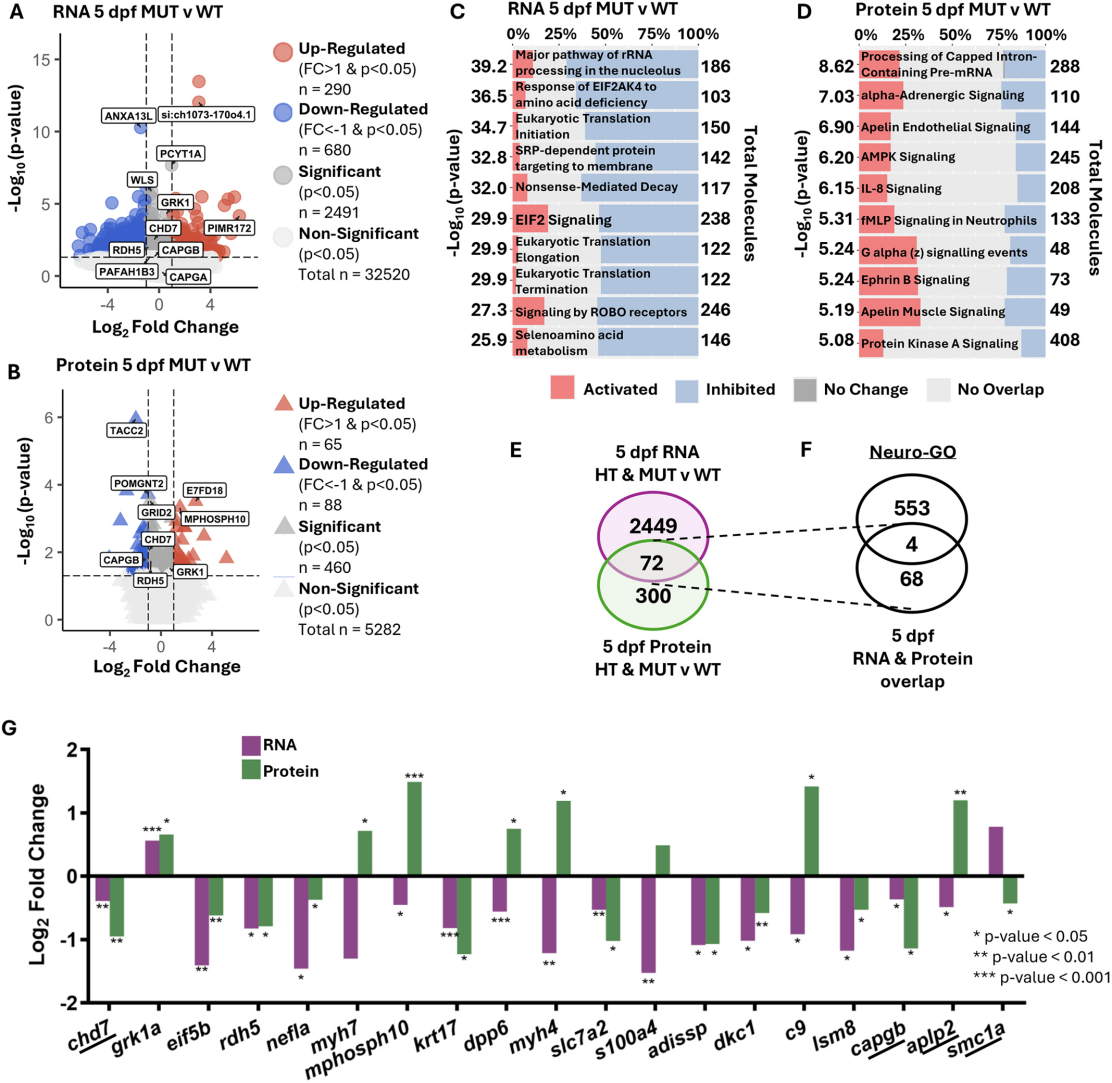
**At 5 dpf, downregulated transcripts with protein counterparts and inhibition of pathways emerge.** (A) Volcano plot of DEGs comparing 5 dpf MUT to 5 dpf WT samples. (B) Volcano plot of DEPs comparing 5 dpf MUT compared to 5 dpf WT samples. (C) IPA pathway enrichment by patterns of DEGs from 5 dpf MUT compared to WT samples. (D) IPA pathway enrichment by patterns of DEPs from 5 dpf MUT compared to WT samples. (E) Overlap of DEGs with *P*<0.05 (Wald test) from 5 dpf HT and MUT versus WT samples (purple), and DEPs with *P*<0.05 (one-way ANOVA) from 5 dpf HT and MUT versus WT samples (green). (F) Overlap of 5 dpf DEGs and DEPs from E and Neuro-GO list. (G) Log_2_ FC for selected DEGs and DEPs from overlap in E and F (underlined). *P<0.05, **P<0.01, ***P<0.001.

To identify Chd7 target genes that may mediate zebrafish CHARGE-like behavioral phenotypes that emerge at 5 dpf, we compared 5 dpf transcriptome and proteome data from *chd7* HT and MUT to WT samples. We identified a set of 3263 DEGs and 56 DEPs in *chd7* HT samples and 2491 DEGs and 460 DEPs in *chd7* MUT samples (Fig. 4A,B; Fig. S13). Homozygous loss of *chd7* at 5 dpf caused twice as many DEGs to be downregulated than upregulated (680 downregulated versus 290 upregulated) (Fig. 4A), and we saw a similar pattern at the protein level, with 88 downregulated and 65 upregulated proteins (Fig. 4B). We next used IPA to uncover molecular pathways that are dysregulated in the absence of *chd7* based on DEGs (Fig. 4C; Fig. S14, Tables S14 and S15) and DEPs (Fig. 4D; Fig. S14, Tables S16 and S17). At the RNA level, most altered canonical pathways were inhibited rather than activated in *chd7* HT and MUT samples, and multiple RNA regulatory pathways were disrupted in both HT and MUT samples, including EIF2 signaling, nonsense-mediated decay, and eukaryotic translation initiation, elongation and termination (Fig. 4C; Fig. S14). We also observed decreased ROBO receptor signaling in both HT and MUT samples, indicating potential for axon guidance defects. At the protein level, only one canonical pathway was disrupted in both HT and MUT samples – processing of capped intron-containing pre-mRNA (Fig. 4D; Fig. S14) – providing further indication of disruptions to RNA processing with loss of *chd7*.

We next created Venn diagrams to visualize the shared transcripts with protein counterparts that were dysregulated in *chd7* HT and MUT samples (Fig. 4E,F; Figs S15 and S16). We found 72 transcripts with protein counterparts that were significantly different (*P*<0.05) at 5 dpf in either HT or MUT samples (Fig. 4E; Fig. S16). Of those 72, three were upregulated and 39 were downregulated as both transcript and protein. Furthermore, four of the 72 were canonically neuro-related (Fig. 4F; underlined in Fig. 4G): *chd7*, *capgb*, *amyloid beta (A4) precursor-like protein 2* (*aplp2*) and *structural maintenance of chromosomes 1A* (*smc1a*). Of these, *chd7* and *capgb* were consistently downregulated at the RNA and protein levels, whereas *aplp2* and *smc1a* had opposing transcript and protein expression patterns. We looked for additional potential *chd7* targets by prioritizing significance and presence in MUT samples and plotted the expression patterns of 15 more of the 72 genes (Fig. 4G). Of these, *eukaryotic translation initiation factor 5B* (*eif5b*), *rdh5*, *nefla*, *keratin 17* (*krt17*), *solute carrier family 7 member 2* (*slc7a2*), *adipose secreted signaling protein* (*adissp*), *dyskeratosis congenita 1 dyskerin* (*dkc1*) and *LSM8 homolog, U6 small nuclear RNA associated* (*lsm8*) were consistently downregulated at the RNA and protein levels.

These unbiased analyses further confirm the validity of our zebrafish model of CHARGE syndrome in showing that *chd7* is strongly downregulated, and they strengthen the possibility that *capgb*, which was consistently downregulated at 3 and 5 dpf, is an important molecular mediator of Chd7.

### Identification of *chd7*-dependent upstream regulators, functions and potential mediators of CHARGE phenotypes

**Fig. 5.**
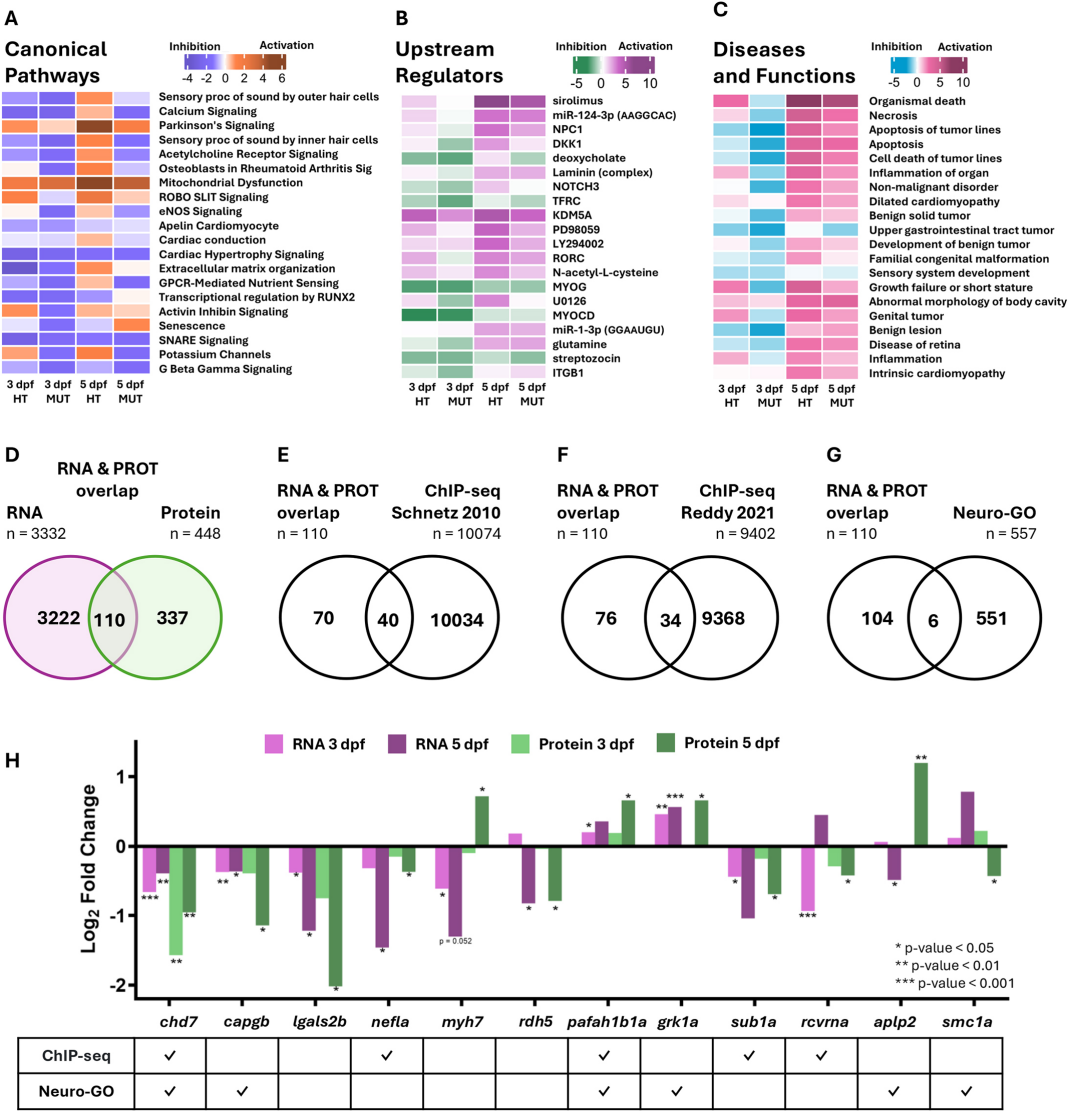
**Transcript and protein expression patterns inform downstream pathway, regulator and functional analysis.** (A) IPA canonical pathway enrichment by patterns of DEGs with *P*<0.05 (Wald test) from 3 dpf and 5 dpf HT and MUT versus WT comparisons. (B) IPA upstream regulator enrichment by patterns of DEGs with *P*<0.05 from 3 dpf and 5 dpf HT and MUT versus WT comparisons. (C) IPA disease and function enrichment by patterns of DEGs with *P*<0.05 (Wald test) from 3 dpf and 5 dpf HT and MUT versus WT comparisons (IPA canonical pathway, upstream regulator, and disease and function enrichment by patterns of DEPs are shown in Fig. S18). (D) Overlap of DEGs with *P*<0.05 (Wald test) from 3 dpf and 5 dpf HT and MUT versus WT comparisons and DEPs with *P*<0.05 (one-way ANOVA) from HT and MUT versus WT comparisons. (E) Overlap of DEGs and DEPs from D with chromatin immunoprecipitation sequencing (ChIP-seq) data from Schnetz et al. (2010). (F) Overlap of DEGs and DEPs from D with ChIP-seq data from Reddy et al. (2021). (G) Overlap of DEGs and DEPs from D with Neuro-GO list. (H) Log2 FC of selected candidate DEGs and DEPs from MUT versus WT comparison plotted at 3 dpf and 5 dpf, and table below noting presence in ChIP-seq or Neuro-GO list. *P<0.05, **P<0.01, ***P<0.001.

To reveal *chd7*-dependent molecular processes from consistent gene expression patterns in HT and MUT samples, we used IPA to further integrate our transcriptomic and proteomic analyses from zebrafish at 3 and 5 dpf. First, we used patterns of DEGs and DEPs to define enriched canonical pathways in HT and MUT larvae (Fig. 5A; Fig. S17, Tables S18 and S19). These canonical pathways, upstream regulators, and disease and function analyses consider patterns of upregulation and downregulation, and, by integrating multiple timepoints and sample comparisons, we identified trends between conditions. DEG data showed consistent disruption of multiple cardiac and neural pathways across genotypes and timepoints: apelin cardiomyocyte, cardiac conduction, cardiac hypertrophy signaling, sensory processing of sound by inner and outer hair cells of the cochlea, calcium signaling, acetylcholine receptor signaling, ROBO-SLIT signaling and SNARE signaling (Fig. 5A). DEP data revealed many fewer altered pathways, most likely due to the smaller set of DEPs identified compared to DEGs (Fig. S17). In 5 dpf MUT samples, however, we also found inhibition of several neural pathways such as CREB signaling in neurons, synaptogenesis signaling, glutaminergic receptor signaling, serotonin receptor signaling and oxytocin in brain (Fig. S17). Next, we identified upstream regulators using IPA by patterns of DEGs and DEPs in HT and MUT samples from 3 and 5 dpf (Fig. 5B; Fig. S17, Tables S20 and S21). Most upstream regulators were inhibited at 3 dpf but activated at 5 dpf based on transcript data (Fig. 5B). Protein data showed that GABA signaling was disrupted at 5 dpf, along with other key neural factors including RB1 and beta-estradiol (Fig. S17). Finally, our analysis of diseases and functions with IPA (Fig. 5C; Fig. S17, Tables S22 and S23) revealed general inhibition at 3 dpf and activation at 5 dpf in both *chd7* HT and MUT samples (Fig. 5C). Several CHARGE syndrome-related functions were altered, such as sensory system development, growth failure, disease of retina and seizure disorder. Together, these findings paint a picture in which loss of *chd7* causes widespread molecular dysfunction that differs at the transcript and protein levels and shifts during development.

The complexity of molecular changes that result from *chd7* loss of function (Figs 1-5) makes it challenging to define individual factors that contribute directly to CHARGE syndrome phenotypes, as they most likely emerge from this collection of changes. However, our datasets highlight some consistent patterns across genotypes and timepoints, which support an effort to isolate genes that may mediate the effects of *chd7* on neural development and behavior. We hypothesized that the most likely candidates would be dysregulated at 3 and 5 dpf, in both HT and MUT samples, at both RNA and protein levels, and would be targets of *chd7*-mediated chromatin accessibility. First, we found 110 genes with protein counterparts that were significantly dysregulated (*P*<0.05) in the 3 or 5 dpf samples (Fig. 5D; Fig. S18). Next, we cross-checked those genes with publicly available ChIP-seq data from mouse embryonic stem cells (Schnetz et al., 2010) and cerebellar granule neurons (Reddy et al., 2021) to find those that *chd7* binds within 3000 bp of the transcription start site (TSS) (Fig. 5E,F; Figs S19 and S20). Finally, we compared these genes with our list neurodevelopmental genes from GO Accession terms and Ensembl (Neuro-GO) (Fig. 5G). These analyses uncovered 11 genes (Fig. 5H), including *chd7*, which was significantly downregulated in all samples (*P*<0.05), binds within 3000 bp of its own TSS and is canonically neuro-related. *capgb* was also downregulated in all samples, is canonically neuro-related, and is expressed in the epidermis and periderm (daniocell.nichd.nih.gov). *Lectin galactoside-binding soluble 2* (*lgals2*) was downregulated in all samples and is expressed in the zebrafish visual system at 60 h post fertilization (hpf) (Thijssen et al., 2006). *nefla* was also downregulated in all samples, and is a Chd7 target by ChIP-seq, has a paralog (*neflb*) known to regulate neuron apoptosis (Wang et al., 2019) and has been found to be expressed in oligodendrocytes (daniocell.nichd.nih.gov). In contrast, *myh7* has opposing transcript and protein expression and is not canonically neuro-related. However, *myh7* is known to be involved in actin filament binding and cardiac development (Hesaraki et al., 2022), is expressed in cardiac muscle (daniocell.nichd.nih.gov) and, thus, may contribute to *chd7*-dependent cardiac development. Similarly, *rdh5* transcript and protein are significantly downregulated at 5 dpf; and although *rdh5* is not canonically neuro-related, it is involved in retinal development, is expressed in the retinal photoreceptor layer and the epiphysis (Thisse and Thisse, 2004), and variants of *RDH5* in humans cause delayed dark adaption (Yamamoto et al., 1999). Finally, *platelet-activating factor acetylhydrolase 1b* (*pafah1b*), upregulated in all samples, is a Chd7 target by ChIP-seq and is canonically neuro-related.

In summary, we reveal six candidate molecular mediators of *chd7* for which dysregulation may cause CHARGE syndrome model phenotypes. Of the six, ChIP-seq data indicate that *nefla* and *pafah1b* are directly regulated by Chd7, and *capgb* and *pafah1b* are canonically neuro-related, while *lgals2*, *nefla*, *myh7* and *rdh5* have been shown previously to be involved in or expressed during nervous system development (Thijssen et al., 2006; Hesaraki et al., 2022; Thisse and Thisse, 2004; Sur et al., 2023).

### Knockdown of candidate genes causes CHARGE syndrome-related behavioral and morphological phenotypes

To determine whether dysregulation of our candidate genes causes behavioral and/or morphological phenotypes consistent with those of our zebrafish CHARGE syndrome model (Hodorovich et al., 2023), we used CRISPR/Cas9 to knock down each gene using two gRNAs targeting different sites in the gene (Table S24). gRNAs were complexed with Cas9 protein and injected into one-cell-stage zebrafish embryos. We raised injected embryos to 5 dpf and recorded, tracked and analyzed behavioral responses of these F0 crispants to acoustic and visual stimuli. We also documented any morphological defects. All injected embryos were screened for gene edits, and those in which one or both target sites were edited were included in the analyses (Fig. S21), with injected but unedited embryos serving as controls (Table S24). Neither gRNA for *pafah1b* or *lgals2* induced edits (Figs S21 and S22), and so we focused on the remaining four candidate genes – *capgb*, *nefla*, *rdh5* and *myh7.* In parallel, we also tested *chd7* WT, HET and MUT larvae to provide context for potential phenocopy in CRISPR-injected fish.

**Fig. 6.**
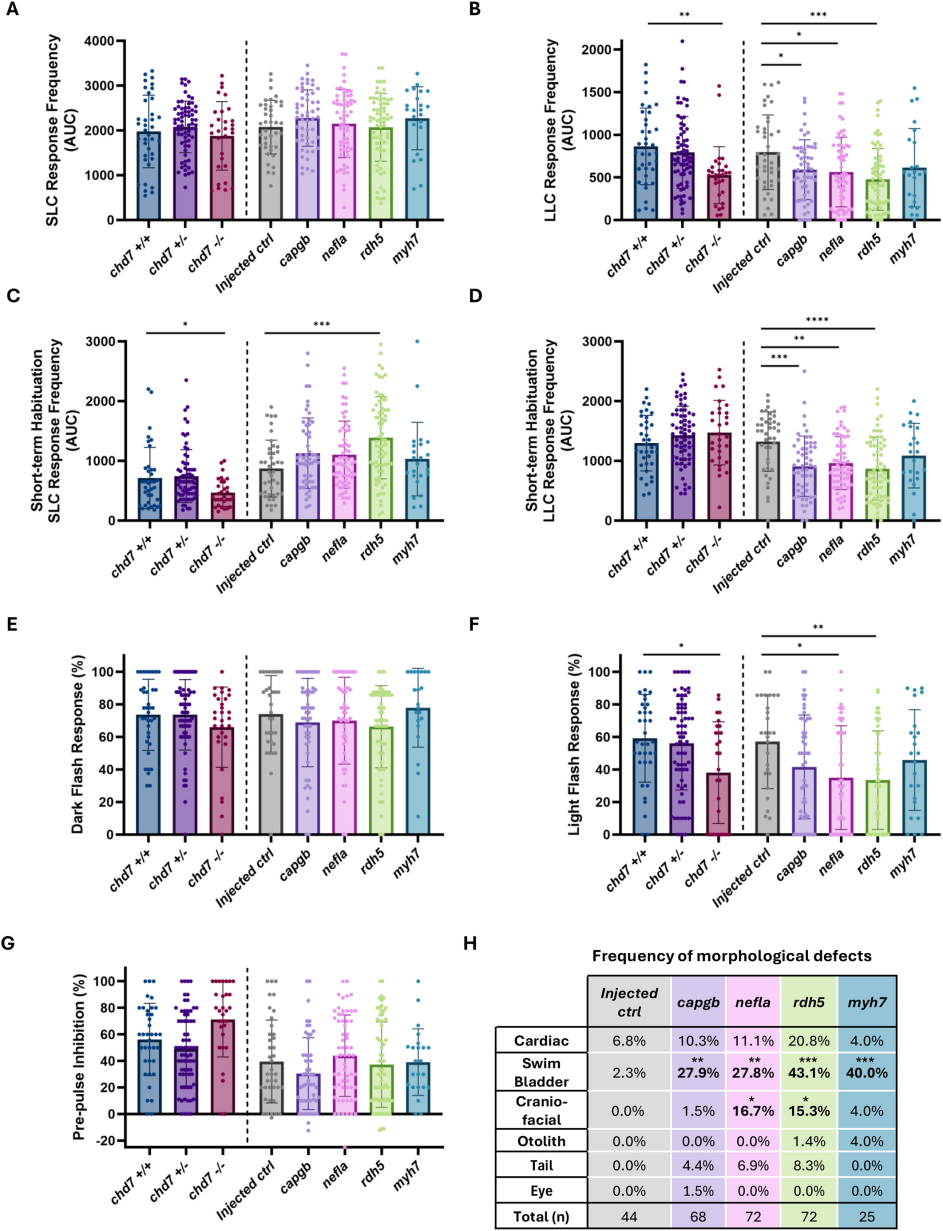
**Knockdown of candidate genes causes phenocopy of CHARGE model phenotypes.** All analyses were performed on larvae derived from multiple clutches in a minimum of two independent experiments. (A) Area under the curve (AUC) plotted for each larva's short-latency C-bend (SLC) response frequency. (B) AUC plotted for each larva's long-latency C-bend (LLC) response frequency. (C) Short-term habituation (STH) AUC for each larva's SLC response frequency. (D) STH AUC for each larva's LLC response frequency. (E) Dark-flash response frequency. (F) Light-flash response frequency. (G) Pre-pulse inhibition percentage. Bar graphs in A-G show mean±s.d. (ordinary one-way ANOVA with Dunnetts's multiple comparisons). *P<0.05, **P<0.01, ***P<0.001, ****P<0.0001. (H) Frequency of morphological defects observed for each group of larvae [Fisher's exact test with Benjamini–Hochberg false discovery rate (FDR) correction]. *FDR<0.05, **FDR<0.01, ***FDR<0.001.

We tested whether sensory processing was affected by knockdown of candidate genes by recording responses of 5 dpf larvae to acoustic stimuli of increasing intensity (Fig. 6A,B; Figs S23 and S24). We found that knockdown of candidate genes *capgb*, *nefla*, *rdh5* and *myh7* did not affect short-latency C-bend (SLC) response frequency (Fig. 6A; Fig. S23). However, long-latency C-bend (LLC) response frequency was reduced upon knockdown of *capgb*, *nefla* or *rdh5* compared to that in injected controls (Fig. 6B; Fig. S24). This phenocopies *chd7* mutant larvae, which also display normal SLC and reduced LLC responses (Fig. 6A,B) (Hodorovich et al., 2023).

We next tested candidate gene crispants for proper habituation to repeated strong acoustic stimulation, a simple form of non-associative learning. Habituation of SLCs was increased by knockdown of *rdh5* (Fig. 6C; Fig. S25). We also found that LLC frequency, which normally increases during habituation, was reduced in *capgb*, *nefla* and *rdh5* crispants compared to that in injected controls (Fig. 6D; Fig. S26). These habituation phenotypes are in contrast to those of *chd7* mutants, which showed faster SLC habituation (Fig. 6C) and no change in LLCs following repeated acoustic stimulation (Fig. 6D). We also tested another form of startle modulation, pre-pulse inhibition (PPI), and found that, similar to *chd7* mutants, knockdown of any of our candidate genes had no effect on PPI (Fig. 6G).

Next, we measured responses to visual stimuli following knockdown of each candidate gene (Fig. 6E,F). All crispants and *chd7* mutants displayed normal O-bend response frequency when given a series of ten dark-flash stimuli (Fig. 6E). However, knockdown of *nefla* or *rdh5* decreased light-flash response frequency compared to that in injected controls, comparable to that seen in *chd7* mutants (Fig. 6F). Finally, we assessed the presence of morphological defects that are seen in *chd7* mutants, including cardiac, swim bladder, craniofacial and otolith defects (Hodorovich et al., 2023). Knockdown of each of the four candidate genes caused an increase in the frequency of observed defects compared to controls, and there was a significant increase in swim bladder defects for all four candidates, and a significant increase in craniofacial defects in *nefla* and *rdh5* crispants (Fig. 6H). Knockdown of *rdh5* resulted in the highest frequency of morphological phenotypes, with 20.8% having cardiac defects, 43.1% having swim bladder defects, 15.3% having craniofacial defects and 1.4% having otolith defects (Fig. 6H). Otolith defects are very rare in clutches of WT embryos but are the most penetrant phenotype in *chd7* mutants (Hodorovich et al., 2023). We saw otolith defects in both *rdh5* and *myh7* crispants, indicating a potential role for both genes in mediating *chd7*-dependent otolith formation.

In summary, knockdown of *capgb*, *nefla* or *rdh5* causes behavioral defects in response to acoustic and visual stimuli similar to those seen in *chd7* mutants. Additionally, loss of any of these four candidate genes results in morphological defects that are also observed in *chd7* mutants. Together, these findings provide evidence that *capgb*, *nefla*, *rdh5* and *myh7* may be novel molecular mediators of Chd7 that contribute to CHARGE syndrome-related developmental and behavioral phenotypes.

## DISCUSSION

The chromatin remodeler CHD7 is critical for the development of many tissues, as evidenced by the widespread deficits seen in patients with CHARGE syndrome, which often include complex behavioral challenges including autism spectrum disorder, sensory processing defects, altered pain sensation, aggression, obsessive compulsive disorder and anxiety (Blake et al., 2005; Edwards et al., 1995; Hartshorne et al., 2005, 2017). The molecular pathophysiology of these neurobehavioral symptoms is not well understood; so, in this study, we sought to uncover and functionally validate molecular mediators of Chd7-dependent neurodevelopment using an established zebrafish CHARGE syndrome model that recapitulates many features of the disorder (Hodorovich et al., 2023). By integrating bulk transcriptomic and proteomic data from two developmental timepoints in *chd7* WT, HT and MUT larvae, we reveal gene expression patterns and canonical pathways that drive *chd7*-dependent neurodevelopment. Finally, using CRISPR/Cas9, we confirmed that multiple candidate genes play roles in zebrafish CHARGE syndrome-related behavioral and morphological phenotypes**.**

CHD7 is a master transcriptional regulator that can enhance or repress transcription (Schulz et al., 2014b). Our data reinforce this concept in that loss of *chd7* does not result in global upregulation or downregulation of transcripts and proteins; but, rather, we observed that *chd7* has bidirectional effects on developmental gene expression. These findings are consistent with transcriptomic analyses of 5 dpf mutant *chd7* zebrafish larval brains (Jamadagni et al., 2021), *Chd7* null mouse embryonic stem cells (Yao et al., 2020), cardiac mouse embryo tissue with conditional knockout of *Chd7* (Stathopoulou et al., 2023) and *Chd7* mutant mouse embryo forebrain tissues (Huang et al., 2025). In contrast, CHD8, a closely related member of the CHD family of chromatin remodelers, seems to primarily enhance transcription as homozygous knockout of *CHD8* in neurons derived from human induced pluripotent stem cells results in markedly more downregulated genes than upregulated genes (Haddad Derafshi et al., 2022). The dual role for *chd7* in both promoting and repressing gene expression adds to the complexity of determining the molecular targets for which dysregulation leads to symptoms of CHARGE syndrome upon *chd7* loss of function.

Proper recruitment of transcription factors and chromatin remodelers such as CHD7 is crucial for regulating gene expression. CHD7 is recruited to enhancers at specific H3K4me1 and H3K4me3 histone modifications and promotes H3K27ac histone modifications by histone acetylases (Lettieri et al., 2021; Reddy et al., 2021). We found enrichment of histone modification signaling (Fig. 3D) and activation of the upstream regulator *lysine demethylase 5A* (*kdm5a*) (Fig. 5B), a histone demethylase that regulates H3K4me3 marks (El Hayek et al., 2020). Similarly, variants in both *CHD7* and *KDM5A* histone-modifying genes are associated with congenital heart disease (Zaidi et al., 2013), and variants in *KDM5A* have been associated with autism spectrum disorder (El Hayek et al., 2020). This is consistent with patients with CHARGE syndrome having behavioral symptoms including autistic-like behaviors and heart defects (Hartshorne et al., 2005; Verloes, 2005). Also, CHD7 interacts with CHD8 (Batsukh et al., 2010), and variants in CHD8 are strongly associated with autism spectrum disorder (Weissberg and Elliott, 2021). Additionally, variants in *KDM6A* are associated with Kabuki syndrome (Van Laarhoven et al., 2015), which has clinical diagnostic criteria that overlap with those for CHARGE syndrome (Schulz et al., 2014a). These findings suggest that loss of *CHD7* may give rise to some behavioral and morphological CHARGE syndrome phenotypes through its role in regulating histone modifications via histone demethylase KDM5A.

The primary focus of this study was the molecular basis of neurodevelopmental deficits caused by loss of *chd7*. Based on patterns of DEGs and DEPs in *chd7* HT and MUT samples, we found enrichment of many neurodevelopmental pathways. One prominently inhibited pathway was calcium signaling (Fig. 3C, Fig. 5A; Fig. S10). Calcium is a key second messenger with many cellular roles including neuronal signaling and plasticity (Rosenberg and Spitzer, 2011), muscle contraction (Fearnley et al., 2011) and apoptosis (Clapham, 2007). We also found inhibition of GABAergic receptor signaling (Fig. S10) and activation of upstream regulator GABA (Fig. S17). It has previously been found that *chd7* is required for GABAergic neuron development via *progestin and adipoQ receptor family member IIIb* (*paqr3b*) regulation in 5 dpf zebrafish (Jamadagni et al., 2021). Although we did not observe significant dysregulation of *paqr3b* in our datasets, our findings further support the role of GABAergic inhibition in *chd7*-mediated neural development and function. We also found impacts of loss of *chd7* on excitatory signaling, with dysregulation of NMDA receptors and postsynaptic events (Fig. S10), synaptic long-term potentiation (Fig. S10) and downregulation of Glutamate receptor ionotropic delta 2 (Grid2) protein (Fig. 4B). Consistent with these findings, *kismet*, the *Drosophila* ortholog of *CHD7* and *CHD8*, has been shown to be important for localization of postsynaptic glutamate receptors and synaptic transmission (Ghosh et al., 2014). GRID2 does not bind glutamate (Araki et al., 1993) but alternatively binds d-serine and glycine (Naur et al., 2007), and has been found to be important for NMDA receptor function (Kumagai et al., 2014) and synaptic plasticity in mouse models (Kakegawa et al., 2011). GRID2 is also expressed in cerebellar Purkinje cells (Araki et al., 1993), in which it has roles in synapse organization (Matsuda et al., 2010). This aligns with our observation of disrupted synaptogenesis signaling in *chd7* HT zebrafish at 3 dpf (Fig. S10). Previously, conditional knockout of *Chd7* in mouse models was found to induce cerebellar hypoplasia and distinct cerebellar foliation anomalies, leading to motor delay and coordination deficits (Whittaker et al., 2017a). Thus, one possibility is that loss of *CHD7* alters cerebellar expression of GRID2, leading to reduced synapse formation and altered excitability, contributing to the cerebellar hypoplasia seen in some patients with CHARGE syndrome (Shiohama et al., 2019).

Our analyses uncovered several additional neurodevelopmental processes affected by loss of *chd7*, including axon guidance, myelination, cytoskeletal regulation and SNARE signaling. Signaling by ROBO receptors (Fig. 4C; Fig. S14) and ROBO-SLIT signaling (Fig. 5A), which are crucial for axon guidance throughout central nervous system development (Brose et al., 1999), were significantly altered in *chd7* HT and MUT samples. This is not the first link between *chd7* and ROBO function, as previous transcriptional analysis identified downregulation of genes associated with ROBO-SLIT signaling in Chd7 mutant mouse heart tissue (Payne et al., 2015). We also observed inhibition of myelination signaling (Fig. 3D), which is consistent with earlier work showing that inactivation of Chd7 causes defects in oligodendrocyte myelination and that Chd7 is required for oligodendrocyte remyelination *in vitro* (He et al., 2016). Our data also highlight multiple cytoskeletal regulatory pathways that were disrupted by loss of *chd7*, including actin cytoskeleton signaling, which we discuss below, and downregulation of *nefla* (Fig. 4G, Fig. 5H). *nefla* is orthologous to human *NEFL* and provides structural support to axons (Yuan et al., 2017). Finally, SNARE signaling was strongly inhibited at both the RNA and protein levels (Fig. 5A; Fig. S10), suggesting that disruptions to the fundamental processes of neurotransmitter release and receptor trafficking contribute to symptoms of CHARGE syndrome. Most of these neurodevelopmental processes were disrupted more strongly at 3 dpf than at 5 dpf, suggesting that *chd7* largely functions to promote the proper formation of neural circuits rather than their function. Together, the data from our zebrafish model provide confirmation of many previous findings regarding the conserved roles of *chd7* in neural development while also setting a foundation for further investigation of the novel mechanisms our analyses have revealed.

Several actin-related processes were affected by loss of *chd7*, including actin cytoskeleton signaling (Fig. 3C; Fig. S10), striated muscle contraction (Fig. 3C; Fig. S10), ABRA signaling (Fig. 3C), RHO GTPase activity (Figs S14 and S17) and downregulation of *capgb* (Fig. 3G, Fig. 4G). *capgb* is orthologous to human *CAPG*, an actin-regulatory protein that is part of the gelsolin family that serves a crucial role in the organization of the actin cytoskeleton (Johnston et al., 1990) and is known to be part of the TYRO protein tyrosine kinase-binding protein (TYROBP) causal network in microglia (Zhang et al., 2013). Proper regulation of actin dynamics is crucial for many cellular processes in which *chd7* is also implicated, particularly in the nervous system, including neurogenesis (Pacheco and Gallo, 2016), cell migration (Okuno et al., 2017), inner ear development (Tilney et al., 1980) and glial development (Sepp and Auld, 2003). These key roles in CHARGE syndrome-related mechanisms and the persistent disruption of *capgb* expression we observed suggest that additional investigation of the relationship between *chd7* and *capgb* is needed.

Deficits in ear morphology, auditory processing and deafness are the most common symptoms seen in patients with CHARGE syndrome (Morgan et al., 1993) and are observed in models of the disorder (Bajpai et al., 2010; Bosman et al., 2005; Patten et al., 2012). For example, in mouse models, haploinsufficiency of *Chd7* and *Sox2* results in reduced otic cell proliferation, severe malformations of semicircular canals, and shortened cochleae with ectopic hair cells (Gao et al., 2024). Conditional deletion of Chd7 in mouse also results in cochlear hypoplasia and complete absence of the semicircular canals and cristae (Hurd et al., 2010). Our zebrafish model also recapitulates some of these defects, including altered morphology of otoliths, calcium crystal structures in the otic vesicles that are vibrated by auditory stimuli and activate hair cells (Baeza-Loya and Raible, 2023), which is the most penetrant morphological phenotype we observed (Hodorovich et al., 2023). Perhaps one of the most striking findings of the current study is that CRISPR/Cas9 knockdown of candidate mediators *rdh5* and *myh7* causes similar defects in otolith morphology (Fig. 6H). The penetrance of these defects is much lower than that seen in *chd7* HT and MUT larvae, however, indicating that otolith morphology defects are likely driven by combined dysregulation of multiple genes with *chd7* loss of function.

We previously found that *chd7* mutant larvae have specific deficits in responding to auditory stimuli with LLCs but not SLCs (Fig. 6A,B) (Hodorovich et al., 2023). This LLC deficit is independent of otolith defects, however, as both *chd7* mutants with and without otolith defects display reduced LLC frequency (Hodorovich et al., 2023). This indicates that additional auditory processing defects drive this behavioral deficit. Our current multi-omic analyses further support this conclusion, as canonical pathway analysis of *chd7* HT and MUT showed inhibition of sensory processing of sound by inner and outer hair cells (Fig. 5A) and inhibition of sensory system development (Fig. 5C). And, based on consistently dysregulated expression across timepoints in *chd7* HT and MUT, we identified and directly tested four potential mediators of *chd7*-dependent neurodevelopment, three of which when knocked down with CRISPR/Cas9 phenocopied the LLC deficit seen in *chd7* mutants (Fig. 6B; Fig. S24). These data provide strong evidence that *capgb*, *nefla* and *rdh5* contribute to regulation of auditory function by *chd7*. That knockdown of just one of these, *rdh5*, resulted in a weakly penetrant otolith defect is consistent with the conclusion that downstream auditory processing is altered by loss of *chd7*.

*capgb* and *nefla*, through their functions in regulating the cytoskeleton, can be linked with multiple aspects of neural development, but *rdh5* is perhaps a more surprising regulator of auditory responses. Rdh5 functions to catalyze the final step in the production of 11-cis retinaldehyde, the universal chromophore in visual pigments (Jang et al., 2001) and contributes to the metabolism of retinoic acid (RA) (Mao et al., 2023), an important morphogen for development of the hindbrain, in which the neural circuits that drive LLC and SLC responses are located. RA signaling has also previously been linked to inner ear development in mouse models of CHARGE syndrome (Micucci et al., 2014). Furthermore, Hox genes are downstream of RA (reviewed in Nolte et al., 2019), and our current pathway analyses of *chd7* mutants uncovered disruption of Hox genes in hindbrain embryogenesis (Fig. 3C). Although *capgb*, *nefla* and *rdh5* crispants phenocopy the *chd7* LLC deficit following non-habituating auditory stimuli, these crispants also showed reduced LLCs during habituation (Fig. 6D), which is in contrast to the increase in LLCs seen during habituation in *chd7* mutants (Fig. 6D; Hodorovich et al., 2023). This difference suggests that *capgb*, *nefla* and *rdh5* likely have differing roles in regulating LLCs depending on stimulus context (habituating or non-habituating), acting in concert with *chd7* to regulate baseline (non-habituating) LLC responses but opposite to *chd7* in circuits driving habituation of auditory responses. These differences may also arise because we targeted one gene at a time rather than the many pathways in combination that occur with loss of *chd7*. Also, crispants are mosaic such that not every cell is expected to harbor a loss-of-function mutation in the target gene, so we may not see complete phenocopy of *chd7* mutants in F0 individuals even if that gene is a direct mediator of the of effects *chd7* on the LLC circuit.

Eye defects and visual impairments are additional common characteristics of patients with CHARGE syndrome that have been investigated in animal models of the disorder (Krueger and Morris, 2022). Chd7 expression in the retina and retinal defects have been observed and characterized in zebrafish and CHARGE syndrome mouse models (Krueger et al., 2023), and our current molecular pathway analyses of zebrafish *chd7* mutants also reflect disrupted visual processing, as pathways of visual phototransduction (Fig. 3C) and disease of retina (Fig. 5C) were significantly enriched. We also found that knockdown of candidate molecular mediators *nefla* and *rdh5* decreased responses to light flashes with no significant difference in dark-flash responses, phenocopying *chd7* mutants (Fig. 6E,F). These visual startle phenotypes in *chd7* mutants are reversed from those in our original study, in which mutants showed reduced dark-flash but normal light-flash responses (Hodorovich et al., 2023). This shift may be due to a change in the genetic background – which our original study found contributes to CHARGE syndrome-like phenotypes (Hodorovich et al., 2023) – as the original mutant line was made and maintained in the WT *Tüpfel long fin* (TLF) strain derived from University of Pennsylvania stocks, and we have since maintained it with Zebrafish International Resources Center (ZIRC)-derived TLFs. Nevertheless, all transcriptome, proteome and behavioral data used for this study were derived from the same generation of *chd7* fish. Thus, although we cannot rule out that *nefla* and *rdh5* act independently of *chd7* in the visual system, the consistency between the molecular and behavioral changes we observed further supports the conclusion that *nefla* and *rdh5* contribute to *chd7*-dependent neural development. Further investigation of how these pathways contribute to vision loss in CHARGE is warranted.

In summary, we have found that loss of *chd7* causes broad dysregulation of neurodevelopmental, sensory system and gene regulatory processes. From this multi-omic analysis, several molecular mediators of behavioral phenotypes of CHARGE syndrome models have emerged. In future work, it will be important to measure the combinatorial effects the candidate effector genes *capgb*, *nefla* and *rdh5* have on auditory and visual responses to determine whether they function in overlapping gene networks. Another key consideration is that *chd7* has tissue-specific roles in regulating gene transcription (Williams et al., 2024); so, tissue-specific manipulation of *capgb*, *nefla* and *rdh5* would help to illuminate their roles in different parts of the nervous system. Finally, our data highlight many additional genes and pathways that were beyond the scope of this investigation that are likely contributors to CHARGE syndrome pathology that are worthy of investigation, such as Grid2, estrogen signaling and Slit-ROBO-mediated axon guidance. Overall, by integrating transcriptomic and proteomic analysis across two key developmental timepoints, we have found that potential molecular targets of Chd7 – *capgb*, *nefla* and *rdh5* –contribute to the behavioral phenotypes of models of CHARGE syndrome. These findings will help to define new genes and pathways regulated by CHD7 that could be potential therapeutic targets to alleviate the neurobehavioral aspects of CHARGE syndrome.

## MATERIALS AND METHODS

### *Danio rerio* husbandry and maintenance

All animal use and procedures were approved by and in accordance with the North Carolina State University (NCSU) Institutional Animal Care and Use Committee (IACUC) guidelines. All *chd7* WT, HT and MUT larvae, and all candidate gene crispants used were of the TLF background. The TLF strain originated from ZIRC stocks. Adult zebrafish were housed at five fish/l density under a 14 h:10 h light:dark cycle at ∼28°C and were fed rotifers, *Artemia* brine shrimp (Brine Shrimp Direct) and GEMMA micro 300 (Skretting).

To generate embryos for larval testing, male and female pairs were placed in mating boxes (Aquaneering) containing system water and artificial grass. 1-2 h into the subsequent light cycle of the following day, embryos were collected and placed into Petri dishes containing 1× E3 embryo medium. Embryos were sorted for fertilization under a dissecting scope at ∼6 hpf and placed into 10 cm Petri dishes with *n*≤65. All embryos were reared in a temperature-controlled incubator at 29°C on a 14 h:10 h light:dark cycle. Each day until testing, a 50-75% media change was performed.

### Zebrafish model of CHARGE syndrome

In this study, we used a previously characterized zebrafish model of CHARGE syndrome (Hodorovich et al., 2023). CRISPR/Cas9 gene editing induced a 7 bp frameshift deletion that leads to a premature stop codon in exon 9 of *chd7*.

### Dissections

Zebrafish were anesthetized at 3 or 5 dpf with ice-cold E3 medium. Dissections were performed under a dissecting microscope with a scalpel and forceps. The scalpel was used to cut diagonally to obtain head, brain and craniofacial tissue, while excluding yolk tissue. The forceps was used to rinse the head tissue in 1× PBS and then place it in RNAlater (Sigma-Aldrich; RNA) or 1× PBS (protein) for long-term storage. The forceps was subsequently used to place the corresponding tail tissue in methanol for genotyping. Genotyping was performed by fixing tail tissue in methanol, followed by DNA extraction using a lysis buffer of 25 mM sodium hydroxide and 0.2 mM EDTA (base solution), heating at 96°C for 30 min and neutralization with 40 mM Tris-HCl. Then that DNA was used in a PCR reaction with GoTaq DNA polymerase (Promega), the recommended master mix (Promega) and primer pair forward, 5′-GATGATGAGCCCTTCAACCCAG-3′ and reverse, 5′-CAGATGGTTTGAGAACGATTGA-3′. PCR reactions were visualized using gel electrophoresis with a 50 bp ladder in which WT bands are 132 bp and MUT bands are 125 bp. Head tissue in RNAlater was placed on ice for 20 min, then at 4°C for 24 h and at −20°C for long-term storage based on the manufacturer’s protocol.

### Transcriptomics

#### RNA isolation

Once genotypes were known, head tissue was pooled based on genotype, 40 heads for 3 dpf and 20 heads for 5 dpf, using a glass Pasteur pipette into a 1.5 ml tube then stored at −20°C until ready for isolation. For RNA isolation, a New England Biolabs Monarch Total RNA miniprep kit and protocol were used following the instructions in ‘Part 1: Sample disruption and homogenization’ and ‘Part 2: RNA binding and elution’ for tissue up to 10 mg. RNA concentration was determined using an Implen NanoPhotometer. Samples were then sent to the NCSU Genomic Sciences Laboratory (GSL) for 250 bp paired-end sequencing on the Illumina Novaseq.

#### RNA-sequencing analysis

Demultiplexed reads were analyzed at the NCSU Bioinformatics Resource Center computing cluster, and SLURM commands can be found at github.com/melody-create/Transcriptomic-Analysis. Quality control was performed with fastqc/0.11.9. Adapter trimming and quality filtering were performed with fastp/0.21.0 (Chen et al., 2018). An indexed reference was built using GRCz11.fna and GRCz11.gtf input (ensembl.org/info/data/ftp/index.html). Paired-end sample reads were aligned with STAR/2.7.9a aligner (Dobin et al., 2013). Differential expression analysis of un-normalized counts was done with DESeq2/1.42.1 (Love et al., 2014). All RNA-sequencing *P*-values were calculated by Wald test in DESeq2/1.42.1.

### Proteomics

#### Protein isolation

Once genotypes were known, head tissue was pooled based on genotype, 20 heads for 3 dpf and 10 heads for 5 dpf, using a glass Pasteur pipette into a 1.5 ml tube then stored at −20°C until ready for isolation. For protein isolation, tissue was suspended in a 100 µl solution of 50 mM ammonium bicarbonate (pH 8.0) containing 1% sodium deoxycholate (SDC; MilliporeSigma). Samples were lysed by probe sonication via two pulses at 20 s per pulse at a 20% amplitude setting. Cellular debris was removed via centrifugation at 10,000 ***g*** for 5 min at 4°C. Supernatant was retained with a pipette on ice and assessed for protein quantification by bicinchoninic acid (BCA) assay using a Pierce BCA Protein Assay Kit. The standard curve was used to determine the protein concentration of each unknown sample.

#### Materials

The following materials were purchased from Thermo Fisher Scientific: 1 M Tris-HCl pH 7.5, 1 M Tris-HCl pH 8, sodium chloride, ammonium bicarbonate (ABC), Pierce™ Mass Spec Grade trypsin, Pierce™ BCA Protein Assay, Pierce™ Quantitative Colorimetric Peptide Assay, liquid chromatography–mass spectrometry (LC/MS) grade water, LC/MS grade acetonitrile, LC/MS grade formic acid and Vivicon 30 kDa molecular mass cutoff filters. Urea, dithiothreitol (DTT) and iodoacetamide were purchased from Bio-Rad. SDC and calcium chloride were from MilliporeSigma.

#### Filter-aided sample preparation

Isolated protein was reconstituted into 50 mM ABC, 1% SDC and a 200 μl aliquot precipitated and rinsed with 800 μl ice-cold acetone. Protein was allowed to precipitate for 30 min at −20°C. After centrifugation, acetone was decanted and removed entirely by vacuum evaporation. Rinsed protein was reconstituted again in 50 mM ABC and 1% SDC, and the concentration was measured by Pierce™ BCA Protein Assay. A 200 μl volume of each sample containing ∼27 µg protein was incubated with 15 μl of 50 mM DTT at 56°C for 30 min. Samples were transferred to Vivicon 30 kDa filters and washed with 8 M urea in 0.1 M Tris-HCl pH 8. Cysteines were alkylated with 64 μl of 200 mM iodoacetamide and incubated at room temperature in the dark for 1 h. Samples were rinsed three times with 2 M urea, 10 mM CaCl_2_ in 0.1 M Tris-HCl pH 8, then three times with 0.1 M Tris-HCl pH 7. All rinsates were discarded. Trypsin protease solution was added to reach a 1:25 trypsin:protein ratio, and samples were incubated overnight at 37°C. Amounts of recovered peptides were quantified by Pierce™ Quantitative Colorimetric Peptide Assay. Samples were evaporated to dryness in a vacuum concentrator and reconstituted in 98% water, 2% acetonitrile, 0.1% formic acid to reach a protein concentration of 0.5 µg/µl.

#### Liquid chromatography–tandem mass spectrometry (LC-MS/MS) analysis

A 2 µl injection was analyzed by reversed phase nano-LC-MS/MS using an Easy-Nano-1200 nanoLC system (Thermo Fisher Scientific) interfaced with an Orbitrap Exploris 480 (Thermo Fisher Scientific) mass spectrometer. The ‘trap and elute’ configuration consisted of a 0.075 mm×20 mm C_18_ trap column with particle size of 3 µm (Thermo Fisher Scientific Accclaim PepMap™ 100, Part #164946) in line with a 0.075 mm×250 mm C_18_ nanoLC analytical column with particle size of 2 µm (Thermo Fisher Scientific PepMap™, Part #ES902). Peptides were eluted using a solvent gradient of water containing 2% acetonitrile, 0.1% formic acid (MPA) and acetonitrile containing 20% water, 0.1% formic acid (MPB). MPB was held at 5% for 2 min, increased to 25% over 100 min, increased to 40% over 15 min, increased to 95% in 1 min and held at 95% for 13 min. Mass spectrometer parameters were set as follows: 2.0 kV positive-ion mode spray voltage, ion transfer tube temperature of 275°C, master scan cycle time of 3 s, scan range of 375-1600 m/z at 120,000 full width at half-maximum resolving power (at 200 m/z), 300% normalized automaic gain control (AGC) target, 120 ms maximum MS^1^ injection time, radio frequency lens of 40%, 15,000 full width at half-maximum resolving power (at 200 m/z) for data-dependent MS^2^ scans, 0.7 m/z isolation window, 30% normalized high-energy collision-induced dissociation collision energy, 100% normalized AGC target, automated maximum injection time and dynamic exclusion applied for 60 s periods.

#### Data interrogation

Raw nanoLC-MS/MS files were processed with Proteome Discoverer 2.5 software (Thermo Fisher Scientific) using *Danio rerio* (Taxon 7955) protein databases obtained from Swiss-Prot (3274 sequences) and TrEMBL (83,386 sequences). A custom contaminants database was included in the searches to identify presence of human keratin and reagent enzyme peptides. Trypsin was designated as the cleavage reagent with hydrolysis sites at the C-terminus of lysine and arginine. A label-free workflow was employed to obtain protein abundance values. The SEQUEST HT search node was set up with the following parameters: maximum of three missed cleavage sites; minimum peptide length of six amino acids; 5 ppm precursor mass tolerance; 0.02 Da fragment mass tolerance; and maximum of four dynamic modifications per peptide, which were oxidation of methionine, N-terminal acetylation, methionine loss and static carbamidomethylation of cysteine. Peptides were validated by Percolator with q-value set to 0.05 and strict FDR set to 0.01. Protein abundances were calculated using all peptides with normalization to total peptide amount. No scaling was performed. Hypothesis testing on replicate comparisons used one-way ANOVA.

#### Transcriptomics and proteomics integration analyses

All transcriptomic and proteomic data was plotted using R packages and tools, and the R code can be found at github.com/melody-create/Transcriptomic-Analysis. PCA was done using DESeq2/1.42.1 (Love et al., 2014) for transcriptomic data and Proteome Discoverer for proteomic data. Heatmaps were made using Complex Heatmap/2.18.0 (Gu et al., 2016, 2022). RNAs and proteins from heatmap slices were analyzed for gene ontology using the PANTHER 19.0 Overrepresentation Test (released 7 August 2024), doi:10.5281/zenodo.16423886 (released 22 July 2025), *Danio rerio* (all genes in database), GO biological process complete, Fisher's exact test and FDR correction (geneontology.org/). Volcano plots were made using EnhancedVolcano/version 1.20.0 (Blighe et al., 2018). Raw data from ©2000-2025 QIAGEN IPA canonical pathways, upstream regulators, and diseases and functions was plotted using ggplot/version 3.5.1 (Wickham, 2016) and Complex Heatmap.

#### ©2000-2025 QIAGEN IPA

Canonical pathway analysis, upstream regulator analysis, and diseases and functions analysis were performed using ©2000-2025 QIAGEN IPA. Fold change and *P*-value data were imported and filtered by *P*<0.05.

#### ChIP-seq data

Data were downloaded from NCBI NIH Gene Expression Omnibus (GEO) GSM558674 (Schnetz et al., 2010) and GSE164360 (Reddy et al., 2021) and analyzed in R using ChIP seeker/1.40.0 (Wang et al., 2022). R code can be found at github.com/melody-create/Transcriptomic-Analysis.

#### Crispant making and analysis

gRNA target sites and primers were selected using the online web tool CHOPCHOP (version 3). Primers were ordered from Integrated DNA Technologies (IDT). Target sites in breeding adult WT TLF fish were sequenced by Sanger sequencing to confirm target. crRNAs for each confirmed target site were purchased from IDT. CRISPR/Cas9 injection cocktails were prepared by combining crRNA, tracrRNA, Cas9 protein (IDT) and Phenol Red. TLF embryos were collected immediately after spawning, 1 nl of injection mix was injected into one-cell-stage embryos. Embryos were raised to 5 dpf, and behavioral and morphological phenotyping was performed. After phenotyping, each larva was genotyped by PCR using primers flanking the target sequence, and resulting reactions were analyzed by gel electrophoresis. If the banding pattern was different from the unedited single band in uninjected samples (multiple bands, smaller or larger bands), these were considered successful edits to the target site.

#### Behavior testing and analysis

5 dpf zebrafish larvae were tested for responses to auditory and visual stimuli as described previously (Hodorovich et al., 2023, 2024). Briefly, larvae were loaded onto an 6×6 acrylic grid with 9 mm diameter wells filled with E3 medium and illuminated from below with infrared light. The grid was attached to an acoustic shaker, which delivered sound stimuli of varying intensities. Light-flash and dark-flash stimuli were delivered with a programmable LED light source mounted above the grid. Acoustic stimuli were delivered with a 20 s interstimulus interval (ISI), with ten trials at each of six intensities. PPI trials consisted of a weak 29.2 dB stimulus 300 ms before a strong 53.6 dB stimulus, with a 20 s ISI between pairs. STH was induced with 30 strong (53.6 dB) stimuli with a 1 s ISI. Ten light-flash and ten dark-flash stimuli were given with a 30 s ISI. A Photron Mini-UX50 high-speed camera captured images at 1000 frames/s at 640×640 pixel resolution. FLOTE software (Burgess and Granato, 2007) was used for unbiased tracking and kinematic analysis to determine behavioral responses. Data were loaded into GraphPad Prism 10.4.2, with which area under the curve, mean and s.d. were calculated.

#### Morphology

Following behavior testing, each 5 dpf zebrafish larva was placed in an individual well of a 24-well plate and anesthetized with tricaine. Larvae were examined with a dissecting microscope at 3× magnification. Each larva was assessed for morphological abnormalities in the otolith, swim bladder, eye, heart, tail, craniofacial and body axis.

## Supplementary Material

10.1242/dmm.052592_sup1 Supplementary information

Table S1. Input: Unique RNAs and Proteins from Figure 1C-D slice 1 only. Output: PANTHER Overrepresentation Test.

Table S2. Input: Unique RNAs and Proteins from Figure 1C-D slice 2 only. Output: PANTHER Overrepresentation Test.

Table S3. Input: Unique RNAs and Proteins from Figure 1C-D slice 3 only. Output: PANTHER Overrepresentation Test.

Table S4. Input: Unique RNAs and Proteins from Figure 1C-D slice 4 only. Output: PANTHER Overrepresentation Test.

Table S5. Input: Unique RNAs and Proteins from Figure 1C-D slice 5 only. Output: PANTHER Overrepresentation Test.

Table S6. List of neurodevelopmental genes generated from GO Accession terms and Ensembl (Neuro-GO).

Table S7. Canonical pathway data from Figure 2C RNA WT 3 dpf v 5 dpf

Table S8. Canonical pathway data from Figure S4 RNA HT 3 dpf v 5 dpf

Table S9. Canonical pathway data from Figure 2D RNA MUT 3 dpf v 5 dpf

Table S10. Canonical pathway data from Figure S10 RNA 3 dpf HT v WT

Table S11. Canonical pathway data from Figure 3C RNA 3 dpf MUT v WT

Table S12. Canonical pathway data from Figure S10 Protein 3 dpf HT v WT

Table S13. Canonical pathway data from Figure 3D Protein 3 dpf MUT v WT

Table S14. Canonical pathway data from Figure S14 RNA 5 dpf HT v WT

Table S15. Canonical pathway data from Figure 4C RNA 5 dpf MUT v WT

Table S16. Canonical pathway data from Figure S14 Protein 5 dpf HT v WT

Table S17. Canonical pathway data from Figure 4D Protein 5 dpf MUT v WT

Table S18. Canonical pathway data from Figure 5A RNA Canonical Pathways

Table S19. Canonical pathway data from Figure S17 Protein Canonical Pathways

Table S20. Canonical pathway data from Figure 5B RNA Upstream Regulators

Table S21. Canonical pathway data from Figure S17 Protein Upstream Regulators

Table S22. Canonical pathway data from Figure 5C RNA Diseases and Functions

Table S23. Canonical pathway data from Figure S17 Protein Diseases and Functions

Table S24. Candidate gene Ensembl Gene ID and Gene Symbol with corresponding number and percentage of CRISPR-Cas9 induced edits at least one gRNA target site, and gRNA target sequence and primers made using CHOPCHOP
