# Supplementary material for: Multi-omic analyses identify molecular targets of Chd7 that contribute to CHARGE syndrome model phenotypes
Source: Dis Model Mech. 2026 Mar 31;19(3):dmm052592. doi: 10.1242/dmm.052592 (PMC13054947; doi:10.1242/dmm.052592)
Supplement: Supplementary information [file dmm-19-052592-s1.pdf]

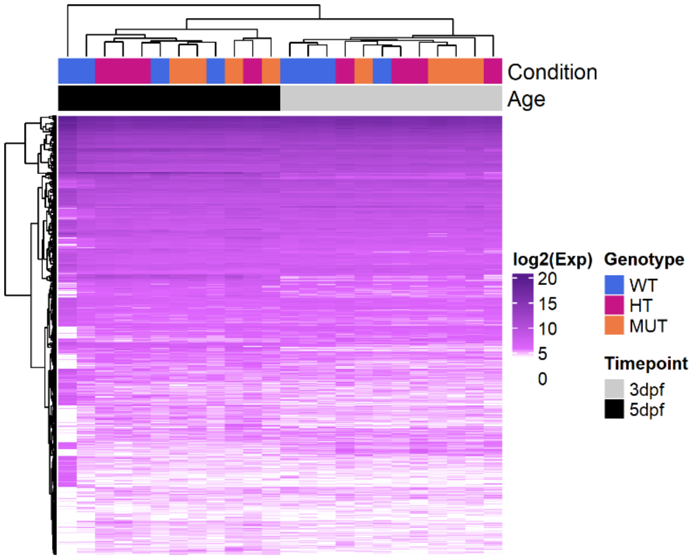

Fig. S1. Heatmap of all biological replicate Log2 Expression with hierarchical clustering by similar expression patterns of Differentially Expressed Genes (DEGs) with a p-value < 0.05 in any comparison and absolute value of Fold Change (FC) greater than 1 (n = 1780).

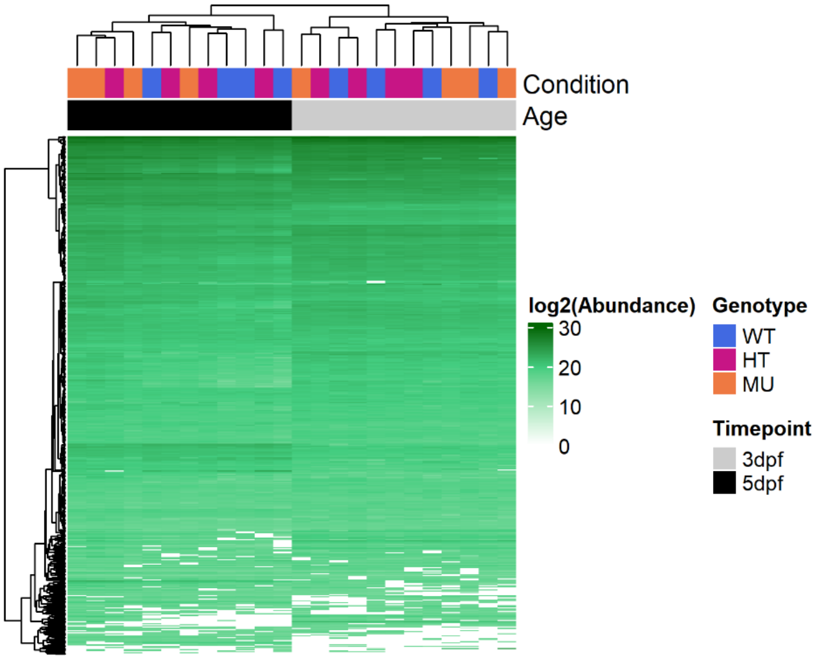

Fig. S2. Heatmap of all biological replicate Log2 Abundance with hierarchical clustering by similar expression patterns of Differentially Expressed Proteins (DEPs) with a p-value < 0.05 in any comparison (n = 553).

Disease Models & Mechanisms • Supplementary information

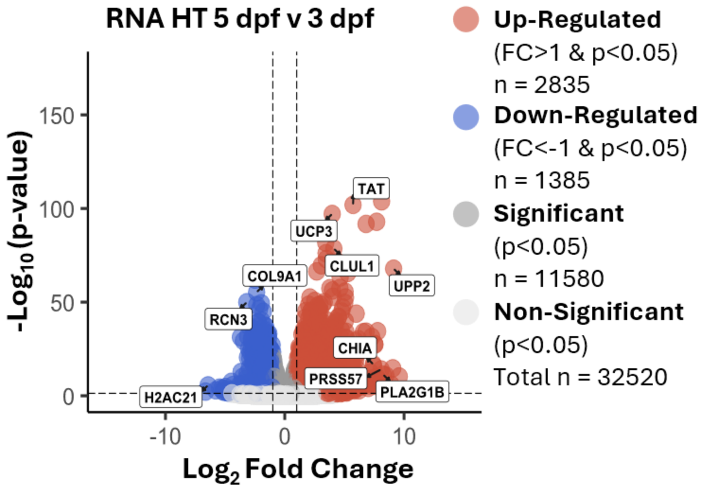

Fig. S3. Volcano plot of Differentially Expressed Genes (DEGs) 5 dpf Heterozygous (HT) compared to 3 dpf HT samples.

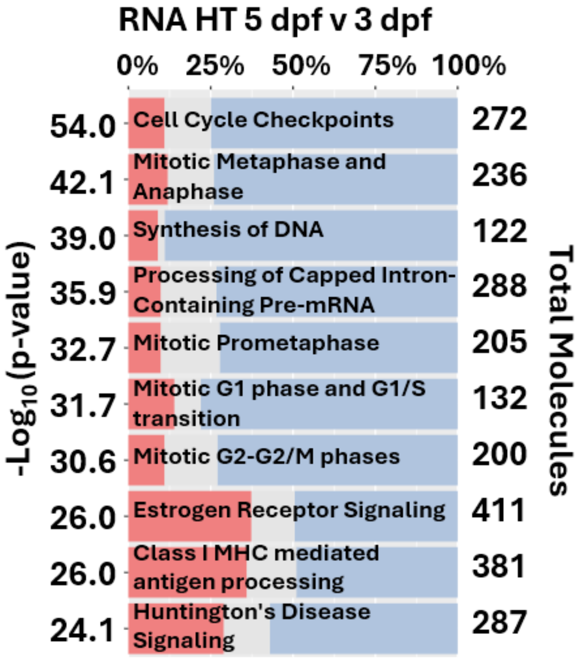

Fig. S4. Ingenuity Pathway Analysis (IPA) pathway enrichment by patterns of DEGs from 5 dpf HT compared to 3 dpf HT samples.

Disease Models & Mechanisms • Supplementary information

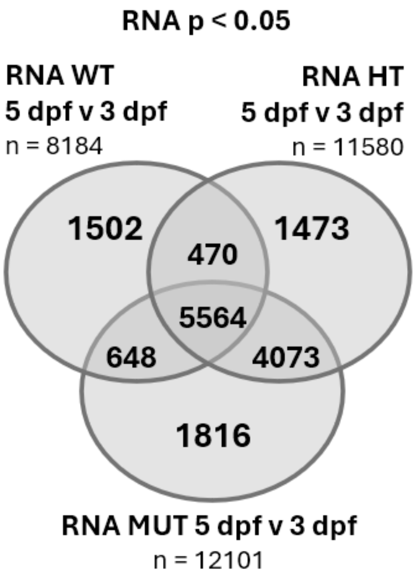

Fig. S5. Overlap of DEGs in WT 5 dpf v 3 dpf, HT 5 dpf v 3 dpf, and MUT 5 dpf v 3 dpf DEGs with p-value < 0.05.

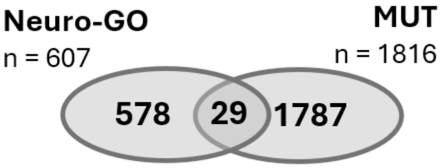

Overlap n = 29

KCNJ2A
ENSDARG00000004459
NPAS4A
NRG2A
OPN7D
SCIN
KCNJ2
SIM1A
CRYGM2D14
ENSDARG00000014854
RGRA
PTN
DIP2BA
TRIM2A
GLRX2
GLRA4A
TRIM3
NRG1
ADGRA2
TAS2R200.1
GSX1
KCNJ10
ARRB1
REL
NEUROD6A
CRYGM2D21
DSCAM
CACNG2A
RGS9B

Fig. S6. List of gene symbol overlap between Neuro-GO list and RNA p < 0.05 MUT 5 dpf v 3 dpf.

Disease Models & Mechanisms • Supplementary information

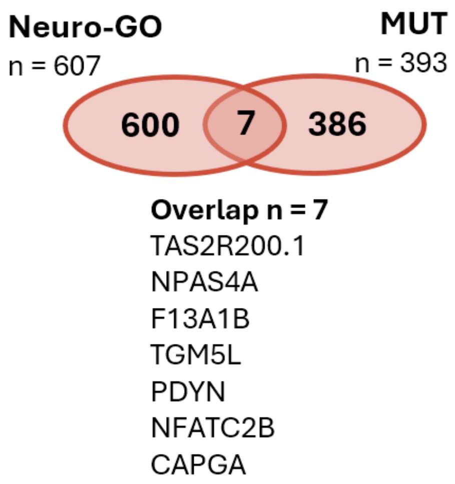

Fig. S7. List of gene symbol overlap between Neuro-GO list and RNA FC > 1 AND p < 0.05 MUT 5 dpf v 3 dpf.

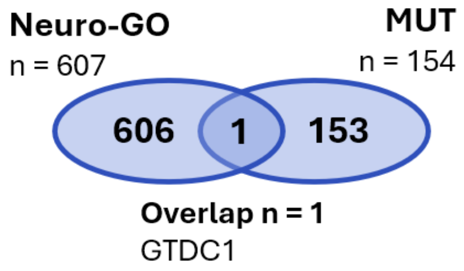

Fig. S8. List of gene symbol overlap between Neuro-GO list and RNA FC < -1 AND p < 0.05 MUT 5 dpf v 3 dpf.

Disease Models & Mechanisms • Supplementary information

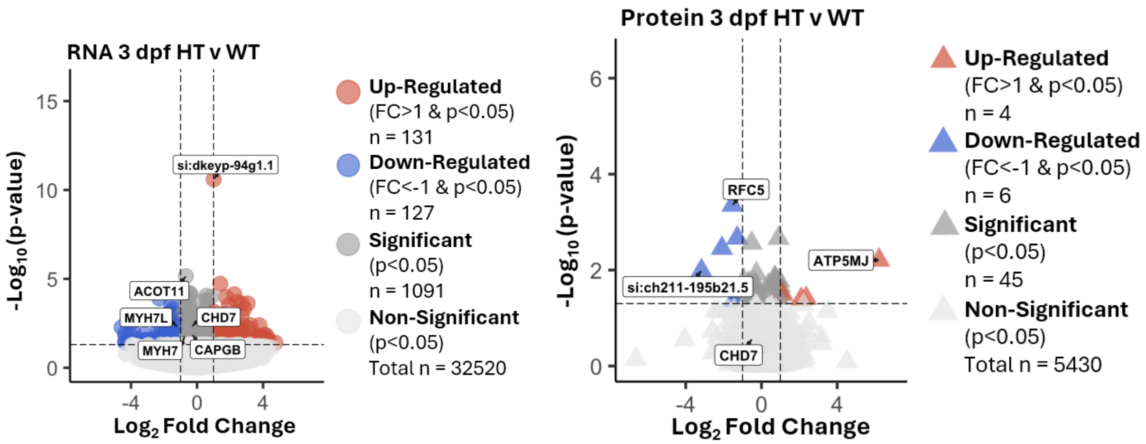

Fig. S9. Volcano plot of Differentially Expressed Genes (DEGs) comparing 3 dpf Heterozygous (HT) compared to 3 dpf Wild Type (WT) samples and Differentially Expressed Proteins (DEPs) comparing 3 dpf HT compared to 3 dpf WT samples.

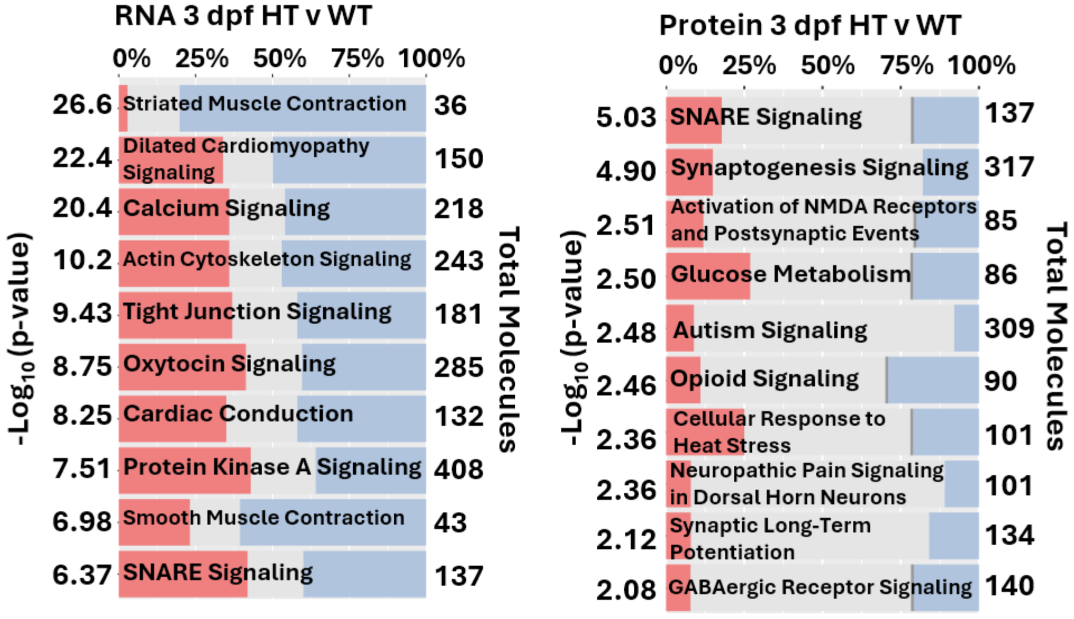

Fig. S10. Ingenuity Pathway Analysis (IPA) pathway enrichment by patterns of DEGs from 3 dpf HT compared to WT samples and by patterns of DEPs from 3 dpf HT compared to WT samples.

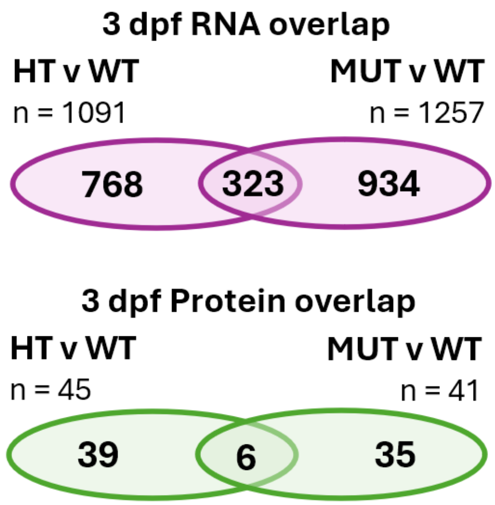

Fig. S11. Overlap of DEGs with p-value < 0.05 from 3 dpf HT v WT and 3 dpf MUT v WT (purple). Overlap of DEPs with p-value < 0.05 from 3 dpf HT v WT and 3 dpf MUT v WT (green).

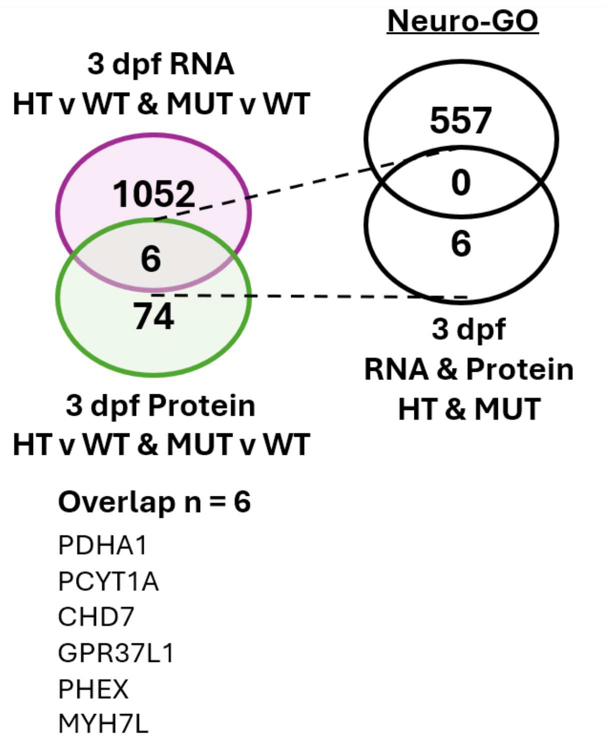

Fig. S12. List of gene symbol overlap between 3 dpf RNA HT v WT & MUT v WT and 3 dpf Protein HT v WT & MUT v WT from Figure 3E. Showing no overlap between Neuro-GO list and 3 dpf RNA & Protein HT & MUT which is the same comparison made in Figure 4F.

Disease Models & Mechanisms • Supplementary information

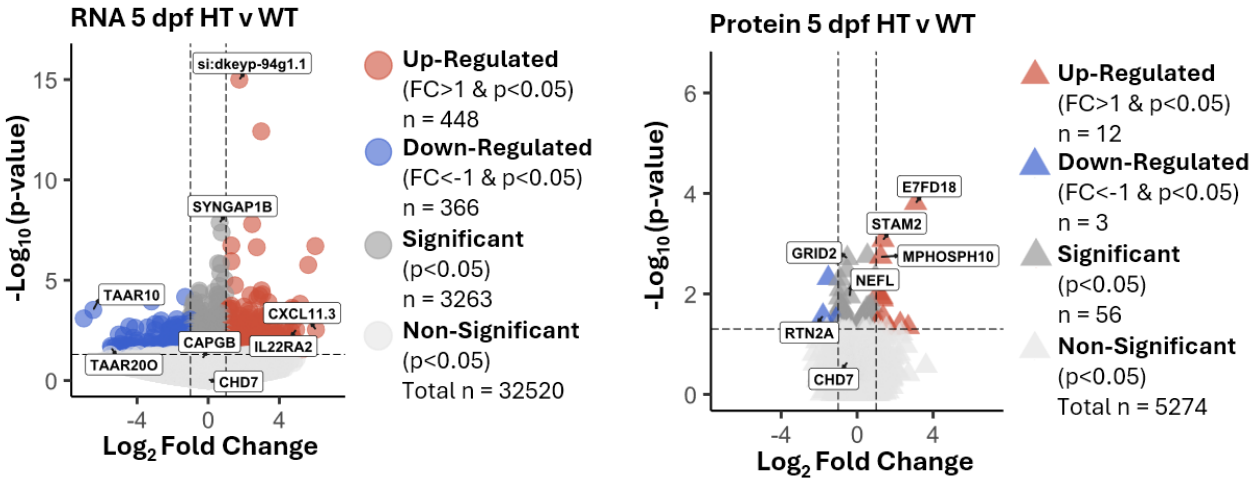

Fig. S13. Volcano plot of Differentially Expressed Genes (DEGs) and Differentially Abundant Proteins (DEPs) comparing 5 dpf Heterozygous (HT) compared to 5 dpf Wild Type (WT) samples.

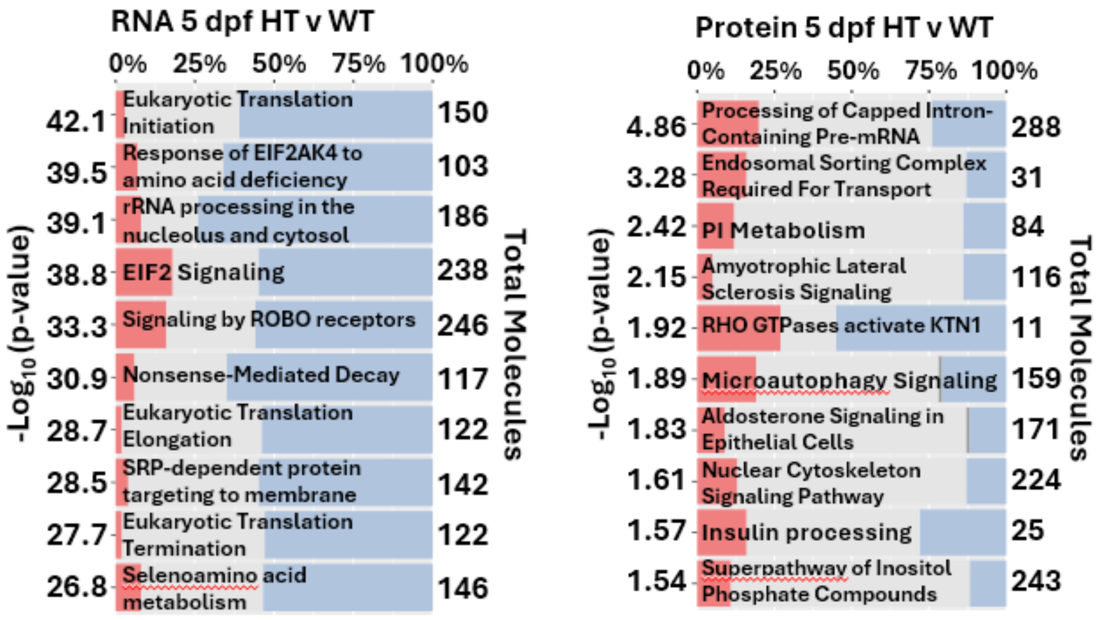

Fig. S14. Ingenuity Pathway Analysis (IPA) pathway enrichment by patterns of DEGs and DEPs from 5 dpf HT compared to WT samples.

Disease Models & Mechanisms • Supplementary information

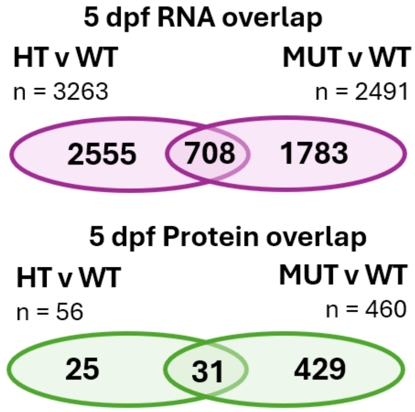

Fig. S15. Overlap of DEGs and DEPs with p-value < 0.05 from 5dpf HT and MUT v WT.

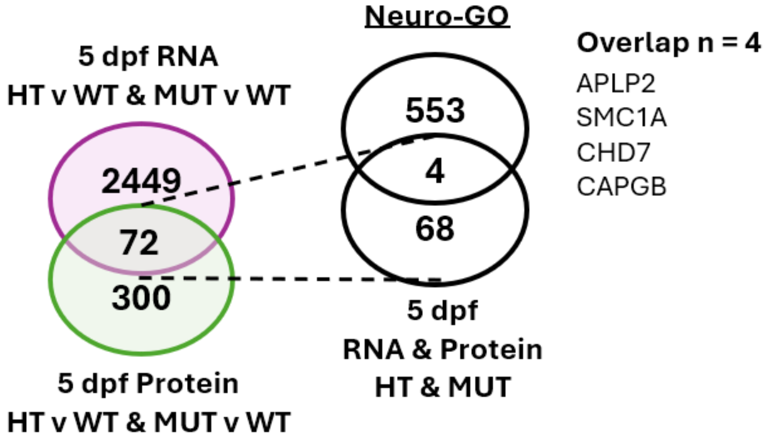

Overlap n = 72

MPHOSPH10	CCDC93	TMEM209
NEFL	CRNKL1	GRK1
PRSS48	MYH4	PHKA2
U2AF2	CAPGB	CTPS1
INPP5K	FUCA2	MBP
SCAMP3	MACF1	CSNK2B
EIF5B	RDH5	SNU13
APLP2	HNRNPAB	SMC1A
S100A4	ARL6IP5	SLC7A2
DKC1	PCBP2	VRK1
CHD7	SEC61A1	COX6B1
HMGB1	GRPEL1	TPM4
HM13	LSM8	SKP1
ABHD16A	RPL22	CD151
CCT8	ADISSP	GSTP1
C9	DYRK1B	RPL24
HSD17B12	EIF2S1	PRDX2
CCT7	RAE1	ANP32E
GMPPA	MAIP1	AK4
ATP6V1G1	MYH7	PSMD4
PFKFB4	WDR3	TVP23B
PSMB2	SLC1A3	DPP6
TXNDC12	NDUFB3	CILP
KRT17	PTCD3	GLRB

Fig. S16. List of gene symbol overlap between 5 dpf RNA HT v WT & MUT v WT and 5 dpf Protein HT v WT & MUT v WT from Figure 4E. List of gene symbol overlap between Neuro-GO list and 5 dpf RNA & Protein HT & MUT from Figure 4F.

Disease Models & Mechanisms • Supplementary information

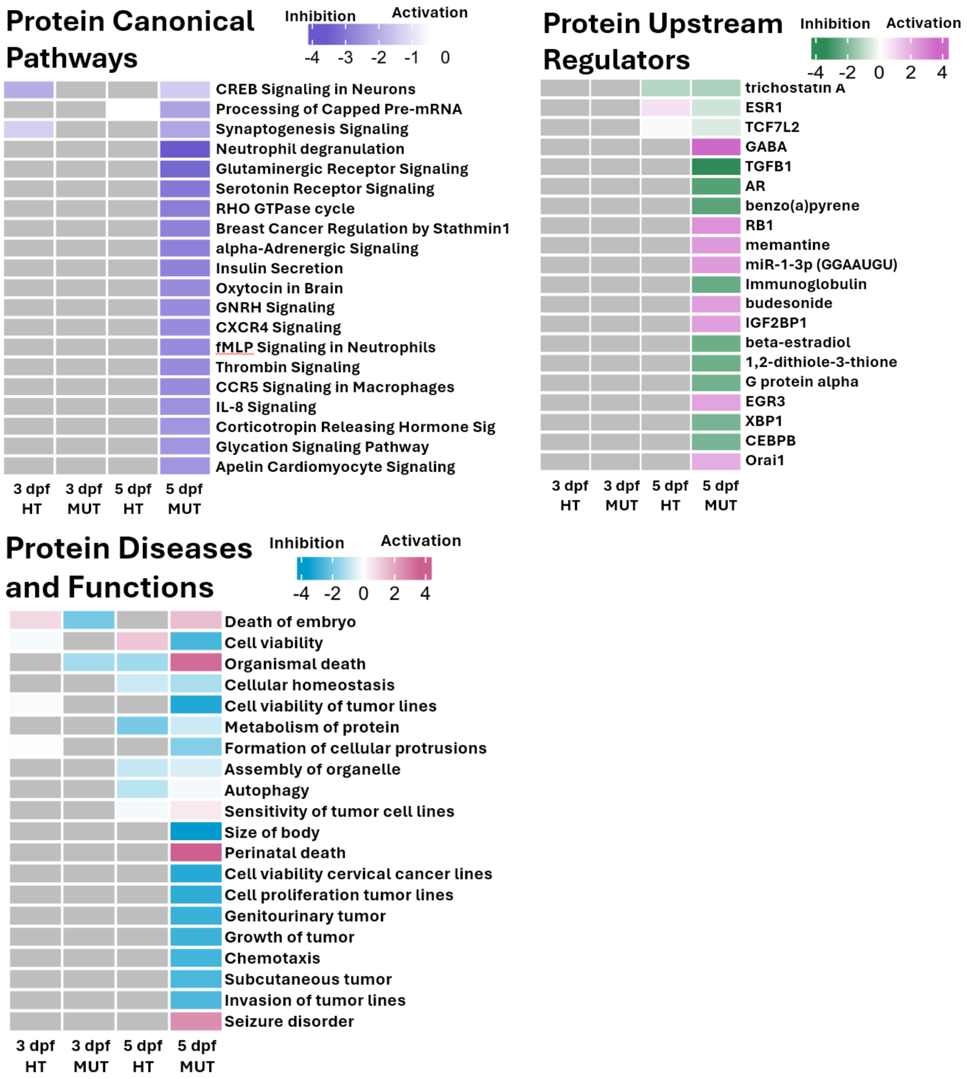

Fig. S17. Ingenuity Pathway Analysis (IPA) canonical pathway, upstream regulator, and diseases and functions enrichment by patterns of DEPs with p-value < 0.05 from 3 dpf and 5 dpf HT v WT and MUT v WT comparisons.

Disease Models & Mechanisms • Supplementary information

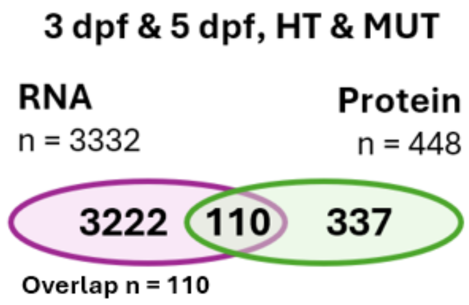

PCYT1A	MYH4	PFKFB4
NEFL	SRCAP	CTPS1
WDR3	S100A4	CILP
CCT8	RPL22	PAFAH1B3
HSD17B12	ANP32E	GMPPA
EIF2S1	CRNKL1	APLP2
SLC1A3	SNU13	MAIP1
RANGAP1	MBP	CAPGB
ABHD16A	ATP5MJ	INPP5K
KRT17	MPHOSPH10	RCVRN
TVP23B	TGFBR3	IL4I1
CD151	HNRNPAB	EPHX1
EIF5B	CCT7	NOL11
U2AF2	MYH7	SLC25A29
DKC1	GLRB	GPR37L1
CSNK2B	HM13	TMEM67
VRK1	HMGB1	PHEX
MACF1	PHKA2	PAFAH1B1
SKP1	SMC1A	GPM6A
FUCA2	DPP6	GSK3A
RAE1	ATP6V1G1	PYGM
NDUFB3	ATP23	NID1
SCAMP3	PDHA1	INTS5
TMEM209	GRK1	SUB1
RPL24	PRSS48	LGALS1
PSMD4	CHD7	MYH7L
ARFRP1	SLC7A2	PRKCE
SEC61A1	RDH5	VWA5A
PSMB2	ADISSP	TSPAN31
ARL6IP5	PRDX2	UNC45B
GRPEL1	AK4	TMOD4
TPM4	FBXO6	STK39
COX6B1	C9	MYBPC3
PCBP2	LSM8	MBOAT2
DYRK1B	TXNDC12	MYBPC2
PTCD3	GSTP1	FREM3
CCDC93	PRKACB	

Fig. S18. List of gene symbol overlap between 3 dpf and 5 dpf, HT and MUT, RNA and Protein from Figure 5D.

Disease Models & Mechanisms • Supplementary information

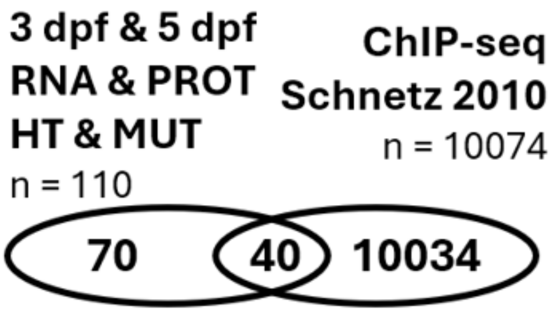

Overlap n = 40

CCDC93	PRKCE
STK39	MBP
ANP32E	NDUFB3
PRKACB	GMPPA
CHD7	GPR37L1
MACF1	HSD17B12
PSMB2	PRSS48
DPP6	ARL6IP5
TGFBR3	MYBPC2
HMGB1	TPM4
TMEM209	CILP
DYRK1B	PHEX
MPHOSPH10	HNRNPAB
GPM6A	TVP23B
FREM3	UNC45B
ATP23	NOL11
SKP1	VRK1
C9	SUB1
SLC1A3	LGALS1
PCYT1A	ABHD16A

Fig. S19. List of gene symbol overlap between 3 dpf and 5 dpf, HT and MUT, RNA and Protein from Figure 5D with public ChIP-seq data from Schnetz 2010 from Figure 5E.

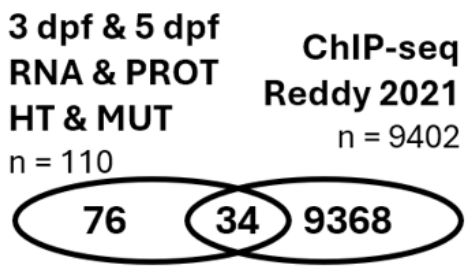

Overlap n = 34

EPHX1
RCVRN
PAFAH1B1
INPP5K
MBOAT2
VRK1
SLC25A29
ATP5MJ
NID1
NEFL
SLC1A3
PCYT1A
MBP
MYBPC3
HSD17B12
GLRB
PRSS48
PRKACB
CHD7
TMEM67
ATP6V1G1
CTPS1
FBXO6
RPL22
DPP6
TGFBR3
HMGB1
LSM8
SEC61A1
COX6B1
SRCAP
PRDX2
PFKFB4
PDHA1

Fig. S20. List of gene symbol overlap between 3 dpf and 5 dpf, HT and MUT, RNA and Protein from Figure 5D with public ChIP-seq data from Reddy 2021 from Figure 5F.

Disease Models & Mechanisms • Supplementary information

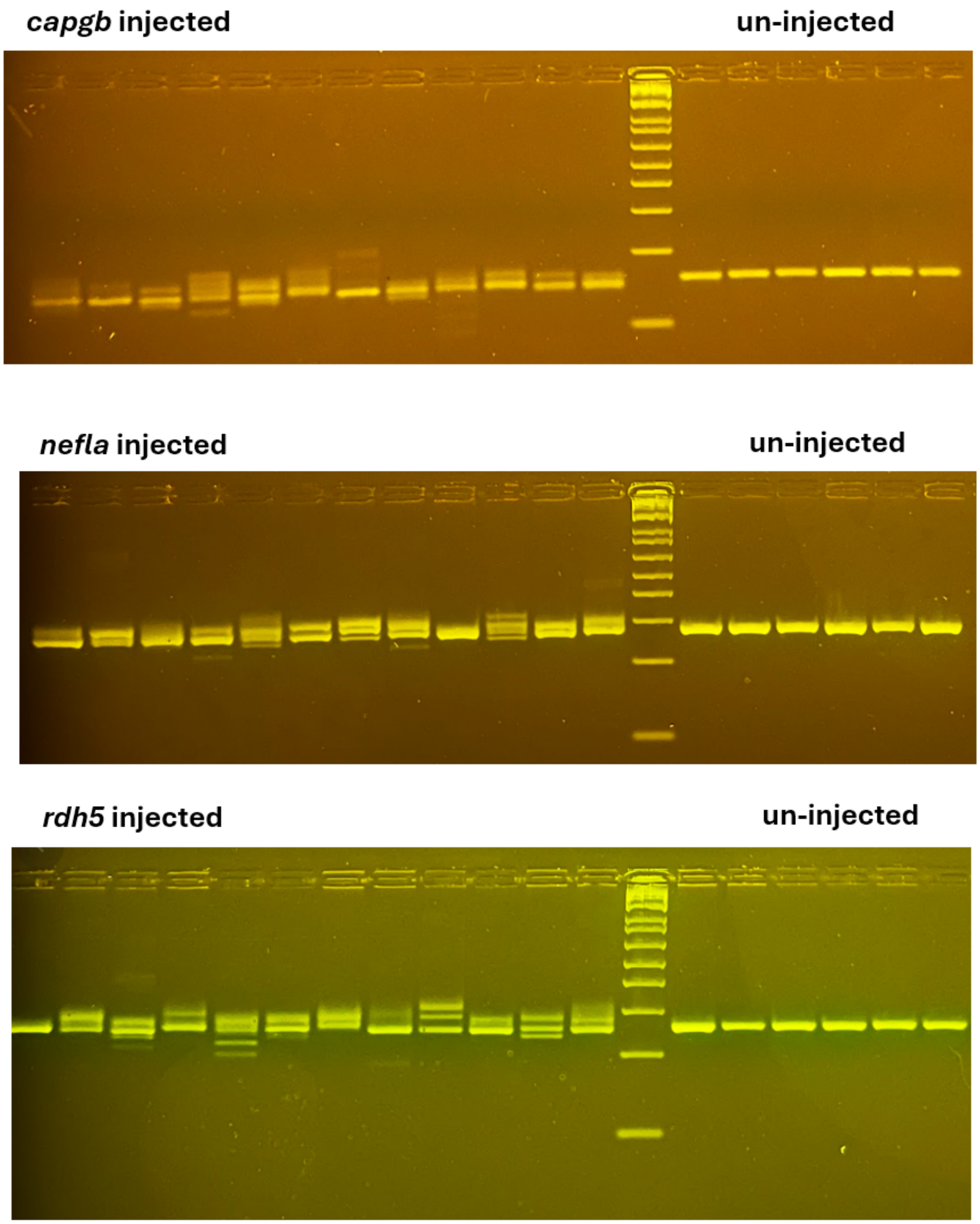

Disease Models & Mechanisms • Supplementary information

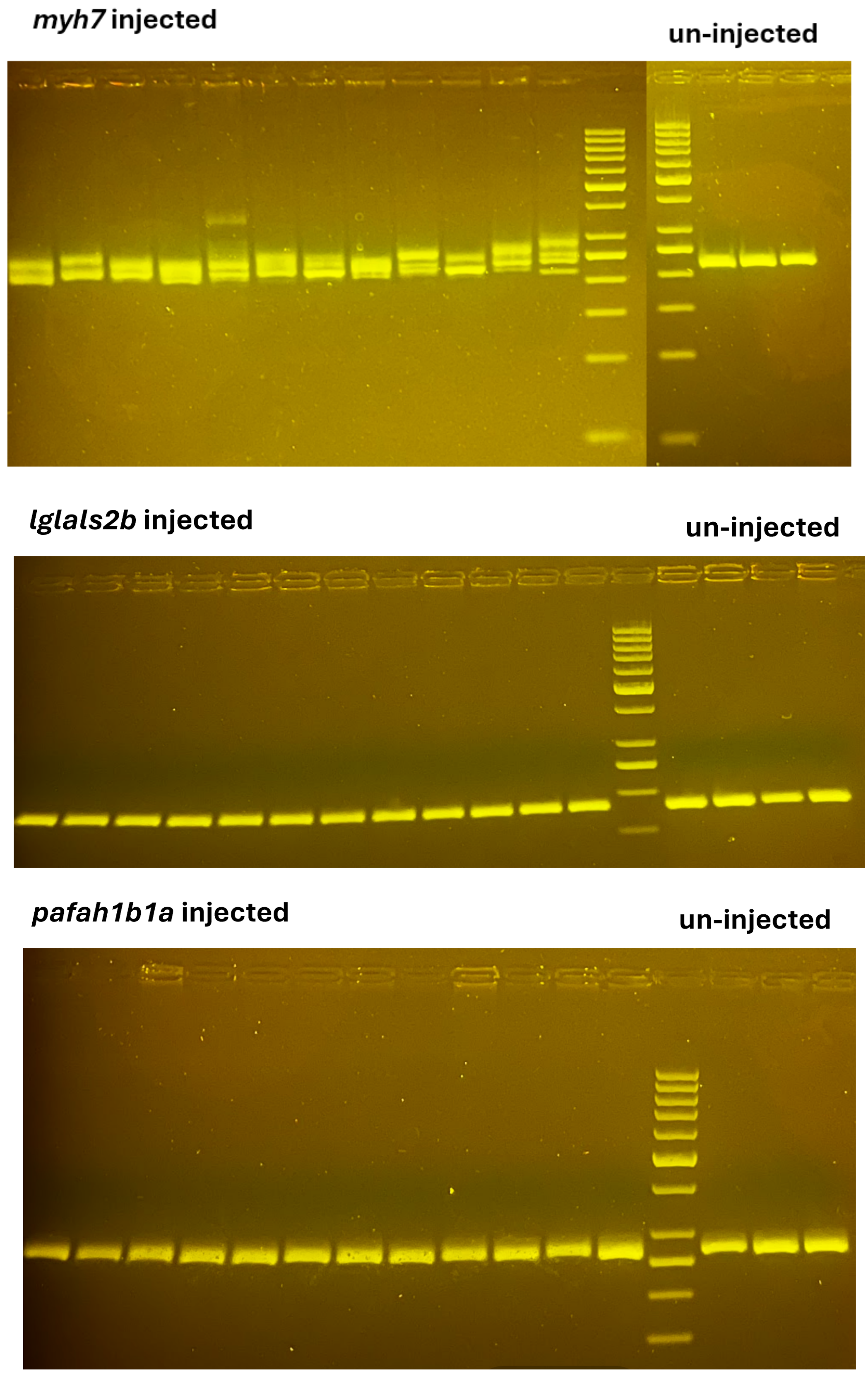

Fig. S21. Confirmation of CRISPR-Cas9 induced edits with designed gRNAs in candidate genes *capgb*, *nelfa*, *rdh5*, and *myh7* and no edits in candidate genes *lglals2b* and *pafah1b1a* by gel electrophoresis of candidate gene gRNA target sites (Table S24).

Disease Models & Mechanisms • Supplementary information

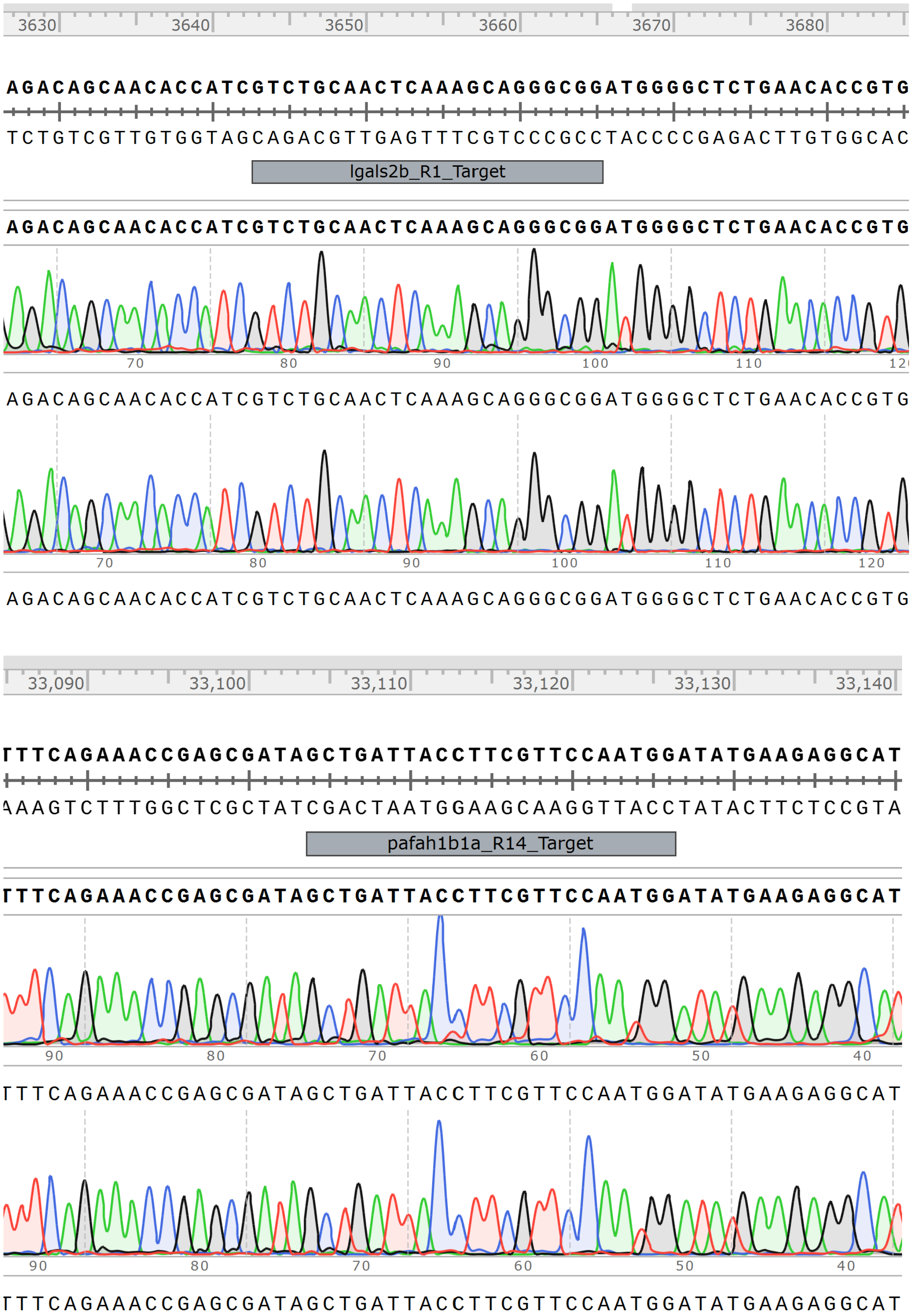

Fig. S22. Confirmation of no CRISPR-Cas9 induced edits with designed gRNAs in candidate genes *lglals2b* and *pafah1b1a* by sanger sequenced gRNA target sites (gray) aligned to Ensembl gene reference sequence, Snapgene version 7.2.1.

Disease Models & Mechanisms • Supplementary information

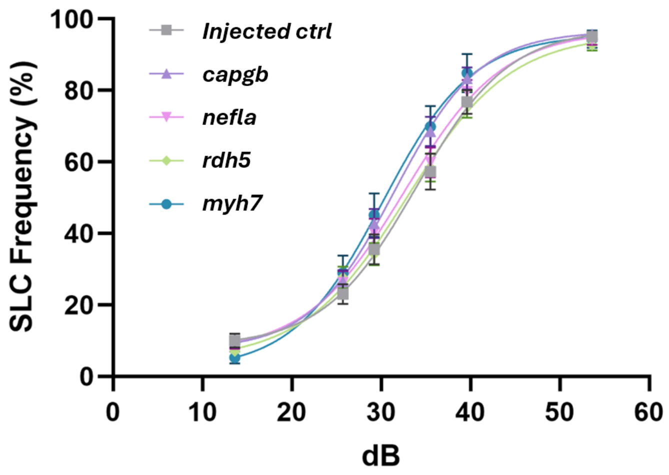

Fig. S23. Short-Latency C-bend (SLC) frequency plotted against acoustic stimulus intensity for each group of larvae fit with a nonlinear regression sigmoidal dose response curve (Mean ±SEM).

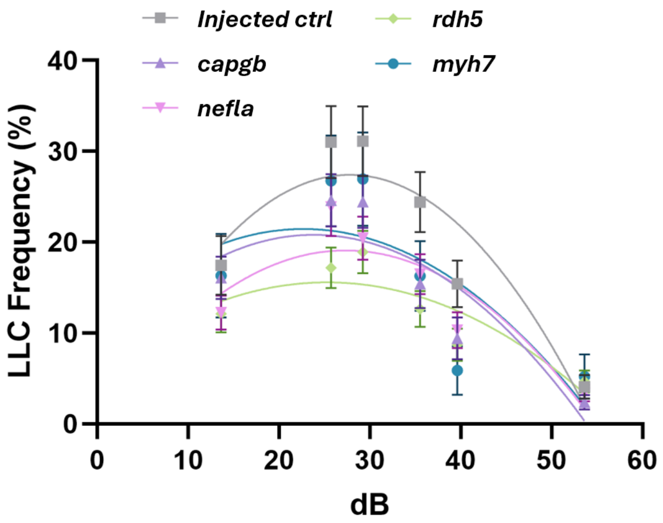

Fig. S24. Long-Latency C-bend (LLC) frequency plotted against acoustic stimulus intensity for each group of larvae fit with a nonlinear regression second order polynomial (quadratic) curve (Mean±SEM).

Disease Models & Mechanisms • Supplementary information

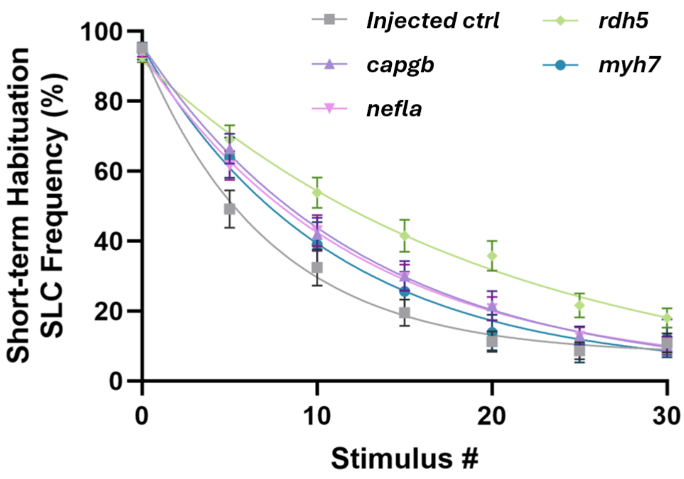

Fig. S25. Short-term Habituation (STH) SLC frequency plotted against time for each group of larvae fit with nonlinear regression one-phase decay curve (Mean±SEM).

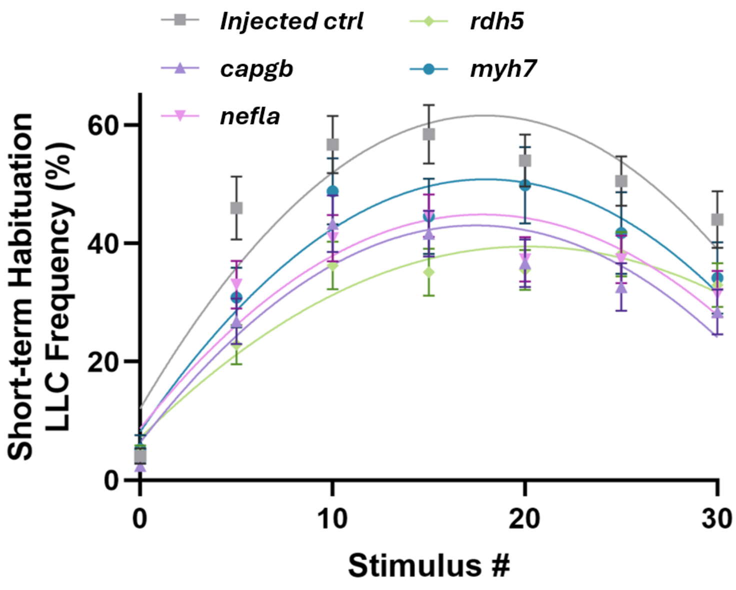

Fig. S26. STH LLC frequency plotted against time for each group of larvae fit with a nonlinear regression second order polynomial (quadratic) curve (Mean±SEM).

Disease Models & Mechanisms • Supplementary information

Table S1. Input: Unique RNAs and Proteins from Figure 1C-D slice 1 only. Output: PANTHER Overrepresentation Test.

Available for download at
https://journals.biologists.com/dmm/article-lookup/doi/10.1242/dmm.052592#supplementary-data

Table S2. Input: Unique RNAs and Proteins from Figure 1C-D slice 2 only. Output: PANTHER Overrepresentation Test.

Available for download at
https://journals.biologists.com/dmm/article-lookup/doi/10.1242/dmm.052592#supplementary-data

Table S3. Input: Unique RNAs and Proteins from Figure 1C-D slice 3 only. Output: PANTHER Overrepresentation Test.

Available for download at
https://journals.biologists.com/dmm/article-lookup/doi/10.1242/dmm.052592#supplementary-data

Table S4. Input: Unique RNAs and Proteins from Figure 1C-D slice 4 only. Output: PANTHER Overrepresentation Test.

Available for download at
https://journals.biologists.com/dmm/article-lookup/doi/10.1242/dmm.052592#supplementary-data

Table S5. Input: Unique RNAs and Proteins from Figure 1C-D slice 5 only. Output: PANTHER Overrepresentation Test.

Available for download at
https://journals.biologists.com/dmm/article-lookup/doi/10.1242/dmm.052592#supplementary-data

Table S6. List of neurodevelopmental genes generated from GO Accession terms and Ensembl (Neuro-GO).

Available for download at
https://journals.biologists.com/dmm/article-lookup/doi/10.1242/dmm.052592#supplementary-data

Table S7. Canonical pathway data from Figure 2C RNA WT 3 dpf v 5 dpf

Available for download at
https://journals.biologists.com/dmm/article-lookup/doi/10.1242/dmm.052592#supplementary-data

Table S8. Canonical pathway data from Figure S4 RNA HT 3 dpf v 5 dpf

Available for download at
https://journals.biologists.com/dmm/article-lookup/doi/10.1242/dmm.052592#supplementary-data

Table S9. Canonical pathway data from Figure 2D RNA MUT 3 dpf v 5 dpf

Available for download at
https://journals.biologists.com/dmm/article-lookup/doi/10.1242/dmm.052592#supplementary-data

Table S10. Canonical pathway data from Figure S10 RNA 3 dpf HT v WT

Available for download at
https://journals.biologists.com/dmm/article-lookup/doi/10.1242/dmm.052592#supplementary-data

Table S11. Canonical pathway data from Figure 3C RNA 3 dpf MUT v WT

Available for download at
https://journals.biologists.com/dmm/article-lookup/doi/10.1242/dmm.052592#supplementary-data

Table S12. Canonical pathway data from Figure S10 Protein 3 dpf HT v WT

Available for download at
https://journals.biologists.com/dmm/article-lookup/doi/10.1242/dmm.052592#supplementary-data

Table S13. Canonical pathway data from Figure 3D Protein 3 dpf MUT v WT

Available for download at
https://journals.biologists.com/dmm/article-lookup/doi/10.1242/dmm.052592#supplementary-data

Table S14. Canonical pathway data from Figure S14 RNA 5 dpf HT v WT

Available for download at
https://journals.biologists.com/dmm/article-lookup/doi/10.1242/dmm.052592#supplementary-data

Table S15. Canonical pathway data from Figure 4C RNA 5 dpf MUT v WT

Available for download at
https://journals.biologists.com/dmm/article-lookup/doi/10.1242/dmm.052592#supplementary-data

Table S16. Canonical pathway data from Figure S14 Protein 5 dpf HT v WT

Available for download at
https://journals.biologists.com/dmm/article-lookup/doi/10.1242/dmm.052592#supplementary-data

Table S17. Canonical pathway data from Figure 4D Protein 5 dpf MUT v WT

Available for download at
https://journals.biologists.com/dmm/article-lookup/doi/10.1242/dmm.052592#supplementary-data

Table S18. Canonical pathway data from Figure 5A RNA Canonical Pathways

Available for download at
https://journals.biologists.com/dmm/article-lookup/doi/10.1242/dmm.052592#supplementary-data

Table S19. Canonical pathway data from Figure S17 Protein Canonical Pathways

Available for download at
https://journals.biologists.com/dmm/article-lookup/doi/10.1242/dmm.052592#supplementary-data

Table S20. Canonical pathway data from Figure 5B RNA Upstream Regulators

Available for download at
https://journals.biologists.com/dmm/article-lookup/doi/10.1242/dmm.052592#supplementary-data

Table S21. Canonical pathway data from Figure S17 Protein Upstream Regulators

Available for download at
https://journals.biologists.com/dmm/article-lookup/doi/10.1242/dmm.052592#supplementary-data

Table S22. Canonical pathway data from Figure 5C RNA Diseases and Functions

Available for download at
https://journals.biologists.com/dmm/article-lookup/doi/10.1242/dmm.052592#supplementary-data

Table S23. Canonical pathway data from Figure S17 Protein Diseases and Functions

Available for download at
https://journals.biologists.com/dmm/article-lookup/doi/10.1242/dmm.052592#supplementary-data

Table S24. Candidate gene Ensembl Gene ID and Gene Symbol with corresponding number and percentage of CRISPR-Cas9 induced edits at least one gRNA target site, and gRNA target sequence and primers made using CHOPCHOP

Available for download at
https://journals.biologists.com/dmm/article-lookup/doi/10.1242/dmm.052592#supplementary-data
